# Comparative Genomic Assessment of Novel Broad-Spectrum Targets for Antibacterial Drugs

**DOI:** 10.1002/cfg.411

**Published:** 2004-06

**Authors:** Thomas A. White, Douglas B. Kell

**Affiliations:** 1 Department of Biology University of York Box 373 Heslington York YO10 5YW UK; 2 Department of Chemistry UMIST Faraday Building Sackville St, PO Box 88 Manchester M60 1QD UK

## Abstract

Single and multiple resistance to antibacterial drugs currently in use is spreading, since they act against only a very small number of molecular targets; finding novel targets for anti-infectives is therefore of great importance. All protein sequences from three pathogens (*Staphylococcus aureus, Mycobacterium tuberculosis* and *Escherichia coli* O157:H7 EDL993) were assessed via comparative genomics methods for their suitability as antibacterial targets according to a number of criteria, including the essentiality of the protein, its level of sequence conservation, and its distribution in pathogens, bacteria and eukaryotes (especially humans). Each protein was scored and ranked based on weighted variants of these criteria in order to prioritize proteins as potential novel broad-spectrum targets for antibacterial drugs. A number of proteins proved to score highly in all three species and were robust to variations in the scoring system used. Sensitivity analysis indicated the quantitative contribution of each metric to the overall score. After further analysis of these targets, tRNA methyltransferase (trmD) and translation initiation factor IF-1 (infA) emerged as potential and novel antimicrobial targets very worthy of further investigation. The scoring strategy used might be of value in other areas of post-genomic drug discovery.


**Comparative and Functional Genomics**
*Comp Funct Genom* 2004; **5**: 304–327.
Published online in Wiley InterScience (www.interscience.wiley.com). **DOI:** 10.1002/cfg.411

**Research Article**

### Comparative genomic assessment of novel broad-spectrum targets for antibacterial drugs

Thomas A. White[1] and Douglas B. Kell[2]*
[1] *Department of Biology, University of York, Box 373, Heslington, York YO10 5YW, UK*
[2] *Department of Chemistry, UMIST, Faraday Building, Sackville St, PO Box 88, Manchester M60 1QD, UK*

**Correspondence to: Douglas B. Kell, Department of Chemistry, UMIST, Faraday Building, Sackville Street, PO Box 88, Manchester, M60 1QD, UK. E-mail: dbk@umist.ac.uk*

Received: 24 November 2003
Revised: 24 March 2004
Accepted: 1 April 2004

### Abstract

**Single and multiple resistance to antibacterial drugs currently in use is spreading, since they act against only a very small number of molecular targets; finding novel targets for anti-infectives is therefore of great importance. All protein sequences from three pathogens (*Staphylococcus aureus, Mycobacterium tuberculosis* and *Escherichia coli* O157:H7 EDL993) were assessed via comparative genomics methods for their suitability as antibacterial targets according to a number of criteria, including the essentiality of the protein, its level of sequence conservation, and its distribution in pathogens, bacteria and eukaryotes (especially humans). Each protein was scored and ranked based on weighted variants of these criteria in order to prioritize proteins as potential novel broad-spectrum targets for antibacterial drugs. A number of proteins proved to score highly in all three species and were robust to variations in the scoring system used. Sensitivity analysis indicated the quantitative contribution of each metric to the overall score. After further analysis of these targets, tRNA methyltransferase (trmD) and translation initiation factor IF-1 (infA) emerged as potential and novel antimicrobial targets very worthy of further investigation. The scoring strategy used might be of value in other areas of post-genomic drug discovery. Copyright © 2004 John Wiley & Sons, Ltd.**

**Keywords:** **genomics; antibacterial; antimicrobial; pathogen; virulence; comparative genomics; antibiotics; bioinformatics**

### Introduction

Within two decades of the introduction of penicillin, the majority of the existing classes of antibacterial drugs had been discovered by systematic screening of natural product libraries. Remarkably, no new chemical classes of active antibacterial drugs were successfully introduced for a further 30 years (Hancock and Knowles, 1998). Table 1 shows the very restricted set of modes of action of the major antibacterial drugs currently in use.

Microorganisms have also shown themselves to be extremely versatile in overcoming the effects of antibacterial drugs. Bacteria have developed a variety of resistance mechanisms and lateral gene transfer mechanisms allow this resistance to be passed between different bacterial strains and species (Davies, 1994; Heinemann, 1999). Antibacterial resistance has developed steadily as new agents have been introduced, and the past 10–15 years have shown a dramatic increase in the occurrence of resistant populations of microbes in both community and hospital environments (Struelens, 1998).

Measures such as chemical modification of existing antibacterial drugs and the development of inhibitors of resistance genes will have a significant impact on antibacterial therapy in the short term. However, it is obvious that new drug targets need to be found if the use of antibacterial

Copyright © 2004 John Wiley & Sons, Ltd.

**Table 1.** Mode of action of the principal established antibacterial drugs

| Drug/class | Function inhibited | Molecular target |
|---|---|---|
| $\beta$-Lactams | Peptidoglycan synthesis | Transpeptidases and carboxpeptidases |
| Bacitracin | Peptidoglycan synthesis | Undecaprenyl pyrophosphate |
| D-Cycloserine | Peptidoglycan synthesis | D-alanine racemase and D-alanyl-D-alanine synthetase |
| Fosfomycin | Peptidoglycan synthesis | UDP-*N*-acetylglucosamine enolpyruvyl transferase |
| Glycopeptides | Peptidoglycan synthesis | Cell wall peptidoglycan |
| Quinolones | DNA replication/transcription | Gyrase and topoisomerase IV |
| Rifamycins | Transcription | RNA polymerase |
| Aminoglycosides | Protein synthesis | 30S ribosomal subunit |
| Chloramphenicol | Protein synthesis | 50S ribosomal subunit |
| Fusidic adid | Protein synthesis | Elongation factor G |
| Macrolides | Protein synthesis | 50S ribosomal subunit |
| Oxazolidinones | Protein synthesis | 50S ribosomal subunit |
| Streptogramins | Protein synthesis | 50S ribosomal subunit |
| Tetracyclines | Protein synthesis | 30S ribosomal subunit |
| Mupirocin | Charging of isoleucyl tRNA | Isoleucyl tRNA synthetase |
| Sulphonamides | Folate synthesis | Dihydropteroate synthetase |
| Trimethoprim | Folate synthesis | Dihydrofolate reductase |

After Chopra *et al.* (2002).

drugs is to continue successfully (Schmid, 1998). To this end, genomic approaches are providing a new strategy by revealing new molecular targets that are giving rise to novel antibacterial agents (Allsop and Illingworth, 2002; Dougherty *et al.*, 2002; Haney *et al.*, 2002; Isaacson, 2002; Ji, 2002; McDevitt and Rosenberg, 2001), as these new agents are unlikely to face the current problems of established mechanisms of resistance (McDevitt and Rosenberg, 2001). In anti-infective research, the inevitable selection for resistant strains means that drugs with multiple targets may be preferred (e.g. multiple penicillin-binding proteins or multiple forms of two-component systems; Stephenson and Hoch, 2002; Stephenson and Hoch, 2004). In other pharmaceutical areas it is encouraging that the rational utility of traditional targets is being confirmed by systematic knock-out studies (Zambrowicz and Sands, 2003).

With the release of data from numerous sequencing projects, the number of potential drug targets has increased massively. However, not all of these molecules will become drug targets (Hopkins and Groom, 2002), and the big challenge is to select the targets most relevant for a given situation (Terstappen and Reggiani, 2001).

Machine learning methods seek to devise new ideas and hypotheses from more or less unstructured data (Gillies, 1996; Kell and Oliver, 2004; Mitchell, 1997; Mjolsness and DeCoste, 2001). It has been shown that such data-driven strategies can be used to identify novel drug targets (Spaltmann *et al.*, 1999). A number of metrics are chosen which should be properties of a potential drug target, such as essentiality and specificity. Each potential target in a genome of interest is scored for these properties. These scores can be weighted differently to add more or less emphasis to any particular property. This scoring system can be tuned so that targets which have already been identified score highly, showing that the scoring system is capable of identifying useful targets. Previously unidentified genes may also score highly, and these can be prioritized as potential drug targets for further study. The top-scoring gene in the study carried out by Spaltmann *et al.* (1999) on antifungal targets was $\alpha$,$\alpha$-trehalose-phosphate synthase, a gene which had never before been suggested as a potential drug target. This shows that post-genomic research has much to offer in terms of novel target identification (Allsop and Illingworth, 2002; Buysse, 2001; Dougherty *et al.*, 2002; Glass *et al.*, 2002; Haney *et al.*, 2002; Isaacson, 2002; Ji, 2002; Knowles and King, 1998; McDevitt and Rosenberg, 2001; Payne *et al.*, 2001a, 2001b; Willins *et al.*, 2002).

In the present study a number of criteria were chosen on which to characterize proteins as targets. These were suggested by the extensive literature on the subject (see e.g. Alksne, 2002; Allsop and Illingworth, 2002; McDevitt and Rosenberg, 2001;

Copyright © 2004 John Wiley & Sons, Ltd. 

Projan, 2002; Spaltmann *et al.*, 1999; Terstappen and Reggiani, 2001). A full list of the criteria used is given in the Methods section.

### Methods

### Data collection and motives

Data were collected from three pathogenic bacterial species, *Staphylococcus aureus*, *Escherichia coli* O157:H7 EDL993 and *Mycobacterium tuberculosis*. These species were chosen as they represent a broad cross-section of bacterial types. Targets which prove to score well in these three species will probably be good targets across a broad spectrum of pathogens.

The entire set of sequences of proteins encoded by *S. aureus*, *E. coli* O157:H7 EDL993 and *M. tuberculosis* were downloaded from the NCBI website (**http://www.ncbi.nlm.nih.gov/PMGifs/Genomes/micr.html**). Each protein was then characterized by a number of criteria which could then be used to prioritize the most suitable proteins as potential antibacterial targets.

A Perl program carried out most of the characterization automatically (see Figure 1 for an overview). Each protein was parsed to find the gene index (gi) number and name of the protein. If the function of the protein was known, or if a function had been assigned to the protein on the basis of sequence homology, then this was noted.

Each protein was then submitted to a BLAST (Altschul *et al.*, 1990, 1997) search (BLASTp, using default parameters except for an 'expectation value' of 0.01) against a local copy of the SwissProt database (**ftp://ftp.ebi.ac.uk/pub/**). The SwissProt database was used because it is well curated, well annotated, non-redundant, and since entries are easily parseable due to its consistent format. There also exist a large number of associated files and websites which use SwissProt-style codes (for species and gene/protein names). Using SwissProt therefore allows these resources to be integrated easily into the program, thus making efficient automation possible.

The results of each BLAST search were parsed to find how many homologues of this protein existed in bacteria, pathogenic bacteria, eukaryotes, mice and *Lactobacillus*. This was done by comparing the SwissProt species ID code of each hit against a look-up table that listed the classification of the organism (**http://ca.expasy.org/cgi-bin/speclist**). A list of bacteria treated as pathogenic in this study is given in Table 2. Bacteria may or may not act as pathogens, depending on the circumstances and the host, and so the list given here covers a broad range of pathogens but is perhaps not completely comprehensive.

The presence of homologues in mice was considered important not only as this will allow targets which are present in higher organisms to be further down-weighted, but also because further down the line the target's absence in mice will make animal trials more effective. *Lactobacillus* spp. are considered to beneficial or probiotic bacteria, so using this metric might be able to prioritize targets which diminish any unwanted side-effects of a new drug.

The scores of BLAST hits against pathogens were also parsed to find how well conserved a particular gene is amongst pathogens. Obviously a protein that is well-conserved across many pathogens will make a better target for broad-spectrum antibacterial drugs. A high degree of conservation may also mean that mutations in the protein are not tolerated, such that resistance is less likely to emerge. The numbers of identical residues in each pathogenic hit compared to the query sequence were summed and then divided by the number of hits against pathogens. This number was normalized by dividing by the length of the query sequence, to give a ratio of conservation for this protein across pathogens.

The query protein was submitted to BLAST separately against the human genome (protein sequences) (**ftp://ftp.ncbi.nih.gov/genomes/H_sapiens/protein/**) and the number of hits was recorded. The closest hit against a human protein was also recorded, with a ratio of similarity given by the number of positive residue matches (matches where amino acids are identical or have similar biochemical properties) divided by the length of the query sequence. The number of positives was chosen so as to err on the side of caution. Any drug designed against a particular bacterial protein may act just as well against a human protein, even if certain key residues are not identical. Similarity of residues may be enough for activity. This metric was included so that potential targets which were not so similar to human proteins would not be so heavily penalized. Even if a human homologue does exist, it may still be possible (e.g. using

Copyright © 2004 John Wiley & Sons, Ltd. 

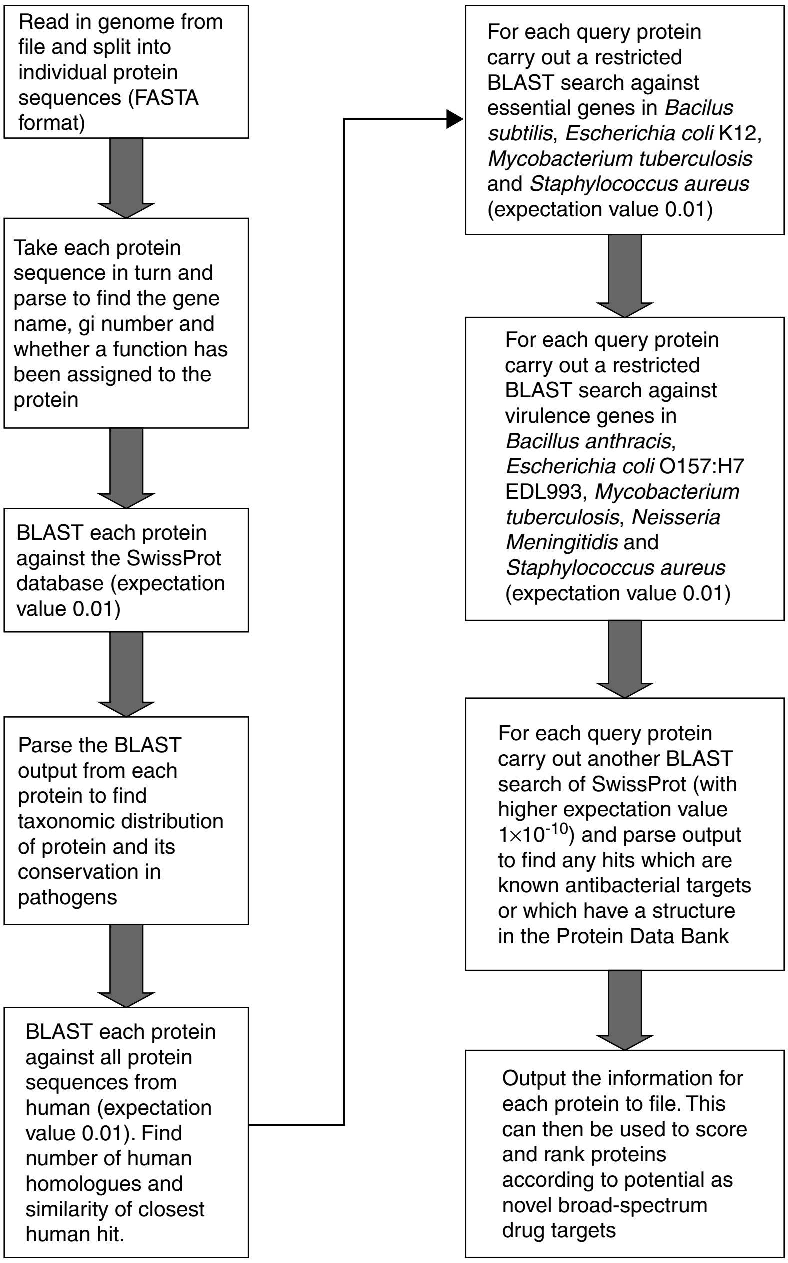

**Figure 1.** Flow chart illustrating the process of data collection structure–activity relationship studies) to design a drug which targets only the bacterial version of the protein.

The query gene was then again submitted to the BLAST program to find homologues which are known antibacterial targets or whose structures have been deciphered. This time an 'expectation value' of $1 \times 10^{-10}$ was used, as to infer suitability as a target or structural similarity it was thought safer to report only very close homologues.

After running the BLAST algorithm, the output was parsed to find whether the query gene was homologous to a known antibacterial target. This was done by comparing the SwissProt gene ID against a list of SwissProt IDs (from the ExPASy website: **http://ca.expasy.org/enzyme/**) of proteins

Copyright © 2004 John Wiley & Sons, Ltd. 

**Table 2.** List of bacteria treated as pathogenic in this study

| | | |
|---|---|---|
| *Acinetobacter calcoaceticus* | *Klebsiella pneumoniae* | *Shigella dysenteriae* |
| *Bacillus anthracis* | *Legionella pneumophila* | *Shigella flexneri* |
| *Bacillus cereus* | *Leptospira interrogans* | *Staphylococcus aureus* |
| *Bordetella pertussis* | *Listeria monocytogenes* | *Staphylococcus aureus* strain Mu50/ATCC 700 699 |
| *Borrelia burgdorferi* | *Moraxella catarrhalis* | *Staphylococcus aureus* strain MW2 |
| *Brucella abortus* | *Moraxella lacunata* | *Staphylococcus aureus* strain N315 |
| *Brucella melitensis* | *Mycobacterium leprae* | *Staphylococcus capitis* |
| *Brucella suis* | *Mycobacterium tuberculosis* | *Staphylococcus epidermidis* |
| *Campylobacter jejuni* | *Mycoplasma fermentans* | *Staphylococcus saprophyticus* |
| *Chlamydia muridarum* | *Mycoplasma genitalium* | *Streptococcus agalactiae* |
| *Chlamydia pneumoniae* | *Mycoplasma hominis* | *Streptococcus agalactiae* serotype III |
| *Chlamydia trachomatis* | *Mycoplasma penetrans* | *Streptococcus agalactiae* serotype V |
| *Clostridium botulinum* | *Mycoplasma pneumoniae* | *Streptococcus mutans* |
| *Clostridium perfringens* | *Neisseria gonorrhoeae* | *Streptococcus pneumoniae* |
| *Clostridium tetani* | *Neisseria meningitidis* | *Streptococcus pyogenes* |
| *Corynebacterium diphtheriae* | *Neisseria meningitidis* serogroup A | *Streptococcus pyogenes* serotype M18 |
| *Enterococcus faecalis* | *Neisseria meningitidis* serogroup B | *Streptococcus pyogenes* serotype M3 |
| *Enterococcus faecium* | *Neisseria meningitidis* serogroup C | *Streptococcus pyogenes* serotype M5 |
| *Escherichia coli* O111:$H^-$ | *Pasteurella multocida* | *Treponema pallidum* |
| *Escherichia coli* O127:H6 | *Propionibacterium acnes* | *Tropheryma whipplei* |
| *Escherichia coli* O157:H7 | *Proteus mirabilis* | *Ureaplasma urealyticum* |
| *Escherichia coli* O6 | *Providencia rettgeri* | *Vibrio cholerae* |
| *Flavobacterium meningosepticum* | *Providencia stuartii* | *Vibrio parahaemolyticus* |
| *Francisella tularensis* | *Pseudomonas aeruginosa* | *Vibrio vulnificus* |
| *Fusobacterium nucleatum* | *Rickettsia conorii* | *Wolinella recta* |
| *Haemophilus ducreyi* | *Rickettsia prowazekii* | *Wolinella succinogenes* |
| *Haemophilus influenzae* | *Salmonella cholerae-suis* | *Xanthomonas maltophilia* |
| *Haemophilus parainfluenzae* | *Salmonella enteritidis* | *Yersinia pestis* |
| *Helicobacter pylori* | *Salmonella typhi* | |
| *Helicobacter pylori* J99 | *Salmonella typhimurium* | |

that are known antibacterial targets (Chittum and Champney, 1995; Egebjerg *et al.*, 1989; Kornder, 2002; Lin *et al.*, 1997; Neu and Gootz, 1996; Schnappinger and Hillen, 1996). Of course, not all current drug targets are perfect examples; indeed, many of the drugs that target them are toxic to humans and resistance has begun to emerge in many cases. Nevertheless, treatments which utilize these targets have been shown to be effective in disease control, and so novel targets possessing similar characteristics to known targets may be useful.

The SwissProt species and protein ID codes of each hit in the BLAST results were compared to a look-up table (**ftp://beta.rcsb.org/pub/pdb/uniformity/derived_data/**) to find out whether any homologues of the query gene had an entry in the PDB database (**ftp://ftp.ncbi.nih.gov/genomes/H_sapiens/protein/**). A protein with a known structure is more attractive from the point of view of further research, as structure-based drug design can be carried out straightaway. A protein with sequence homology to a protein of known structure is likely to have a similar structure (although this is not always true) and so may be favoured as a potential novel drug target.

Each protein was then submitted to several more restricted BLAST searches against selected bacterial genomes. The BLAST searches were restricted by gi number; specifically the gi numbers of genes found to be essential or involved in virulence. These genomes chosen are listed in Table 3.

These genomes were selected as they cover a wide range of bacterial types, and also because they are well characterized and are amongst the few species for which this work has been carried out to any great extent. For those species for which this kind of work has not been done, genomics methods may allow us to predict essentiality or involvement in virulence. Proteins that have significant hits against essential genes or genes involved in virulence are likely to have the same characteristics themselves and so may score highly as potential

Copyright © 2004 John Wiley & Sons, Ltd. 

**Table 3.** List of genomes used for restricted BLAST searches against essential genes or genes involved in virulence

| | |
|---|---|
| Essential genes | |
| Genomes | *Bacillus subtilis* (Kobayashi *et al.*, 2003)<br>*Escherichia coli* K12 (**http://www.shigen.nig.ac.jp/ecoli/pec/About.html**)<br>*Mycobacterium tuberculosis* (Sassetti *et al.*, 2003)<br>*Staphylococcus aureus* (Forsyth *et al.*, 2002) |
| Virulence genes | |
| Genomes | *Bacillus anthracis* (Hoffmaster and Koehler, 1999; Koehler, 2002)<br>*Escherichia coli* O157:H7 EDL993 (Brunder *et al.*, 2001; Sharma and Dean-Nystrom, 2003; Stuber *et al.*, 2003; Wang *et al.*, 2002)<br>*Mycobacterium tuberculosis* (Triccas and Gicquel, 2000)<br>*Neisseria meningitidis* (Sun *et al.*, 2000)<br>*Staphylococcus aureus* (Dunman *et al.*, 2001) |

drug targets. The more 'model' genomes in which the gene is found to be essential, the more likely it is that this gene is indeed essential for the query species, and also has greater potential as a target for a broad-spectrum antibacterial drug.

Having assigned each gene in the query genome values for a number of characteristics, these values could then be weighted, summed and ranked to produce a list of high-priority potential targets. This ranking approach was used instead of a machine learning-based approach, as the 'training set' of known antibacterial targets is very small and not necessarily optimal (see Introduction). While the ranking approach is more subjective, it does allow targets to be prioritized which score better according to our metrics than currently known targets.

### Assigning weights and the robustness of target prioritization

A number of different weighting schemes were tried so that the weighting scheme could be refined to reflect the relative importance of the various metrics. After a weighting scheme was run on the raw data, the scores for each metric could be summed and the total scores of the targets then ranked. The refinement of the weightings was done by carrying out a sensitivity analysis on the metric scores for the top few ranking targets. Sensitivity analysis is more normally used in biology to find the importance, or so-called control coefficients (**http://dbk.ch.umist.ac.uk/mca_home.htm**; Fell, 1996; Heinrich and Schuster, 1996; Kell and Westerhoff, 1986), by which each enzyme controls the flux through a metabolic pathway, but can in fact be used to find the relative importance of any variable which contributes to a total. The equation giving the sensitivity of overall metric $A$ to individual metric $v_i$ is given by (equation 1)

$$C_i^A = \frac{\partial A}{\partial v_i} \cdot \frac{v_i}{A} = \frac{\partial \ln A}{\partial \ln v_i} \quad (1)$$

Here a more discretized sensitivity analysis was done for each target by taking the score of each metric of the target, finding 1% of this score, dividing this number by the total score and multiplying by 100. When this is done for all metrics, these 'contributions' sum to 1. Thus, sensitivity analysis asks, 'By altering the score of one variable by 1%, what percentage change would this induce in the total score?'. These sensitivity analyses could clearly show when some variables were exerting too much or too little influence on the total score and therefore the weights could be optimized accordingly. This novel approach proved very useful in carefully modifying the scoring systems.

Using different weighting schemes also allowed the analysis of how robust a particular high-ranking target was to the weighting scheme. Clearly, a target which scores highly due to having favourable characteristics in one highly weighted metric is less good than one which ranks highly under a number of different scoring systems.

For each of five different scoring systems (Table 4) used on *S. aureus*, *E. coli* O157:H7 EDL993 and *M. tuberculosis* the top 20 ranking targets were recorded. These top 20 lists could then be checked against each other to see whether robust targets had emerged. The top 20 lists were then cross-checked to see whether any targets were robust in all three species (see Table 5). This 'voting' method approach can be seen as combining the output of several weak learners, which is known to be a very effective approach to data mining (Bauer and Kohavi, 1999; Dietterich, 2000; Hastie *et al.*, 2001).

The first scoring system was designed to give most influence to those metrics which were felt to be the most important and least influence to those

Copyright © 2004 John Wiley & Sons, Ltd.

**Table 4.** The five different scoring systems used to test the influence of the scoring system on the ranking of targets

| **Metric** | | **Scoring system** | | | | |
|---|---|---|---|---|---|---|
| | | **1** | **2** | **3** | **4** | **5** |
| Copy number | | 20/No. of copies of gene | 100/No. of copies of gene | Same as 1 | Same as 1 | Same as 1 |
| Distribution of homologues | | (50/No. of homologues in bacteria) × No. of homologues in pathogens | (100/No. of homologues in bacteria) × No. of homologues in pathogens | Same as 1 | Same as 1 | Same as 1 |
| Species distribution | | [(500/89) × No. of distinct pathogens with homologues] − (No. of distinct bacteria with homologues − No. of distinct pathogens) | [(100/89) × No. of distinct pathogens with homologues] − (No. of distinct bacteria with homologues − No. of distinct pathogens) | Same as 1 | Same as 1 | Same as 1 |
| Conservation in pathogens | | Conservation ratio ×200 | Conservation ratio ×100 | Same as 1 | Same as 1 | Conservation ratio ×400 |
| Eukaryotic homologues | | 50/(No. of eukaryotic homologues + 1) | 100/(No. of eukaryotic homologues + 1) | Same as 1 | Same as 1 | Same as 1 |
| Human homologues | | 50/(No. of human homologues + 1) | 100/(No. of human homologues + 1) | Same as 1 | Same as 1 | Same as 1 |
| Proximity to closest human homologue | | 100 − (proximity ratio ×100) | 100 − (proximity ratio ×100) | Same as 1 | Same as 1 | Same as 1 |
| Mouse homologues | | 10 − (No. of mouse homologues + 1) | 100 − (No. of mouse homologues + 1) | Same as 1 | Same as 1 | Same as 1 |
| Lactobacillus homologues | | 10 if absent, 0 if present | 100 if absent, 0 if present | Same as 1 | Same as 1 | Same as 1 |
| Function known | | 50 if yes, 0 if no | 100 if yes, 0 if no | Same as 1 | Same as 1 | Same as 1 |
| Homologous to known target | | 20 if yes, 0 if no | 100 if yes, 0 if no | Same as 1 | Same as 1 | Same as 1 |
| Homologous to essential gene in: | *Bacillus subtilis* | 50 if yes, 0 if no | 100 if yes, 0 if no | Same as 1 | 150 if yes, 0 if no | Same as 1 |
| | *Escherichia coli* K12 | 50 if yes, 0 if no | 100 if yes, 0 if no | Same as 1 | 150 if yes, 0 if no | Same as 1 |
| | *Mycobacterium tuberculosis* | 20 if yes, 0 if no | 100 if yes, 0 if no | Same as 1 | 100 if yes, 0 if no | Same as 1 |
| | *Staphylococcus aureus* | 20 if yes, 0 if no | 100 if yes, 0 if no | Same as 1 | 100 if yes, 0 if no | Same as 1 |
| Homologous to virulence gene in: | *Bacillus anthracis* | 10 if yes, 0 if no | 100 if yes, 0 if no | 50 if yes, 0 if no | Same as 1 | Same as 1 |
| | *Escherichia coli* K12 | 20 if yes, 0 if no | 100 if yes, 0 if no | 100 if yes, 0 if no | Same as 1 | Same as 1 |
| | *Mycobacterium tuberculosis* | 20 if yes, 0 if no | 100 if yes, 0 if no | 100 if yes, 0 if no | Same as 1 | Same as 1 |
| | *Neisseria meningitidis* | 20 if yes, 0 if no | 100 if yes, 0 if no | 100 if yes, 0 if no | Same as 1 | Same as 1 |
| | *Staphylococcus aureus* | 20 if yes, 0 if no | 100 if yes, 0 if no | 100 if yes, 0 if no | Same as 1 | Same as 1 |
| Homologous to PDB entry | | 10 if yes, 0 if no | 100 if yes, 0 if no | Same as 1 | Same as 1 | Same as 1 |

Copyright © 2004 John Wiley & Sons, Ltd.

**Table 5.** The overall top ten ranking targets

| Rank | Gene name/description | Robustness | Total score |
|---|---|---|---|
| 1 | tRNA methyltransferase (trmD) | 15 | 13 391 |
| 2 | UDP-N-Acetylmuramate-L-alanine ligase (murC) | 15 | 13 229 |
| 3 | UDP-N-acetylglucosamine 1-carboxyvinyl transferase (murA)* | 13 | 13 059 |
| 4 | Translation initiation factor IF-1 (infA) | 14 | 13 019 |
| 5 | DNA polymerase III, $\alpha$ chain (dnaE) | 13 | 12 992 |
| 6 | 30S ribosomal protein S4 (rpsD)* | 11 | 12 779 |
| 7 | UDP-N-acetylmuramoylalanine-D-glutamate ligase (murD) | 11 | 12 766 |
| 8 | 50S ribosomal protein L10 (rplJ) | 11 | 12 755 |
| 9 | Chromosomal replication initiator protein (dnaA) | 10 | 12 716 |
| 10 | UDP-N-acetylmuramoylalanyl-D-glutamate-2,6-diaminopimelate ligase (murE) | 9 | 12 573 |

These targets rank highly in all three species used and rank in the top 20s of most of the scoring systems used. Robustness is how many times the gene ranks in the top 20 under five different scoring systems across the three species used, giving a maximum robustness score of 15. Total score is the sum of the scores for this target in all scoring systems across all species used. The maximum possible total score is 24 120.
* Indicates that the gene is a known target of an antibacterial drug (murA is targeted by fosfomycin and rpsD is a target of tetracyclines).

felt to be least important. Homology to essential genes in *M. tuberculosis* and *S. aureus*, and homology to virulence genes in *Bacillus anthracis* were weighted lower than homology to essential and virulence genes in other organisms. This was done to reflect the quality of the data for these organisms, as different methods were used and lists of essential and virulence genes are not always complete.

Under the second scoring system all metrics were weighted equally, so that a maximum score for one metric would be the same as for another. For the other three scoring systems most of the metrics were weighted as under the first system. However, in the third scoring system homology to virulence genes was given greater influence, in the fourth homology to essential genes was given greater weight, and in the fifth the level of conservation of the target in pathogens was given more importance.

### Further investigation of high-scoring targets

Having narrowed down the number of potential drug targets using the methods outlined above, the highest-scoring targets could then be investigated in greater detail. The top genes were again subjected to a BLAST search against the SwissProt database to determine in which pathogens they were present. The databases Genbank, EMBL and DDBJ were also searched via ENTREZ (**http://www.ncbi.nlm.nih.gov/entrez/query.fcgi**) to find whether or not a copy of the query gene existed in a specific pathogen, in case this had been missed by searching only the SwissProt database. PROSITE (**http://us.expasy.org/prosite/**) was also searched to find any conserved motifs not identified by BLAST, which could be used to find more distantly related homologues of the query gene. This approach was able to identify any human sequences which, although not closely related in terms of sequence homology, could be very similar in terms of structure and biochemical properties to the query gene. Multiple sequence alignments and phylogenetic trees were created using ClustalX (Thompson *et al.*, 1997) and Mega2.1 (Kumar *et al.*, 2001). This was done to determine how distinct the genes in these pathogens were from those homologues in non-pathogens and eukaryotes, and just how well the 'active sites' of these genes were conserved across the different pathogenic species. The available literature was also searched to gain more insights into these suggested targets.

### Results and discussion

### Scores

According to the scoring systems used, the majority of genes in *S. aureus*, *E. coli* O157:H7 EDL993 and *M. tuberculosis* would make very poor antibacterial targets (see Figures 2–4). In all three bacteria there are also only a few high-scoring genes. The highest ranking of these seem to be fairly robust and tend to rank in the top 20, regardless of which scoring system is used (see Table 5).

It is also apparent there are no targets which are perfect in every way. To obtain a perfect score in the present metrics a target should be present

Copyright © 2004 John Wiley & Sons, Ltd. 

in one copy, be present in all pathogens but not in non-pathogens, eukaryotes or humans. It should be perfectly conserved across all pathogens. Its function should be known, it should be homologous to a known target, homologous to essential and virulence genes in all the model genomes used and its structure should be known. Here even the highest-ranking targets achieve only some 50% of the perfect score.

This is perhaps discouraging, as it means that there is little possibility for the development of a 'magic bullet' drug that is highly effective, specifically targets only pathogens, is easy to develop and is immune to the problems of emerging resistance. However, this never was a likely prospect.

The unusual peaks in the distribution graphs are due to genes of unknown function that, when submitted to BLAST with an expectation value of 0.01, did not return any hits. The two peaks in *E. coli* O157:H7 EDL993 target scores occur for the same reason, except that the peak at the higher score is due to genes that return no hits but have been assigned some sort of function, presumably by other methods.

### Scoring systems and sensitivity analysis

The first scoring system used was designed to reflect what is thought to be most important in terms of the properties an antibacterial drug target should possess. Hence, the first scoring system rates as very important the properties 'species distribution', 'conservation in pathogens', 'similarity to human' and 'homology to essential genes'. A target which does not perform well on any one of these criteria will probably not make a good drug target. The emphasis accorded to these properties means that targets which are not present in a wide range of pathogens, are not well conserved, are very similar to targets in humans, or are not essential will not be able to score highly and thus will not be prioritized. The metric 'species distribution' is weighted so that a target will receive the maximum score if it is present in all the bacteria treated as pathogenic by this study and in no non-pathogens. It is unlikely that this maximum would ever be awarded to a target, and so this property is given a very high weighting to compensate for this fact. The other useful properties a target may possess are, in a sense, bonuses and are scored to reflect this. A target does not necessarily need to be (directly) involved in virulence in order for a drug to neutralize an infection. However, involvement in virulence may bring benefits to using a target, in that the target should be absent from most non-pathogens and also absent from humans. The existence of homologues in humans does not matter *per se*; rather, it is the similarity (or lack) of

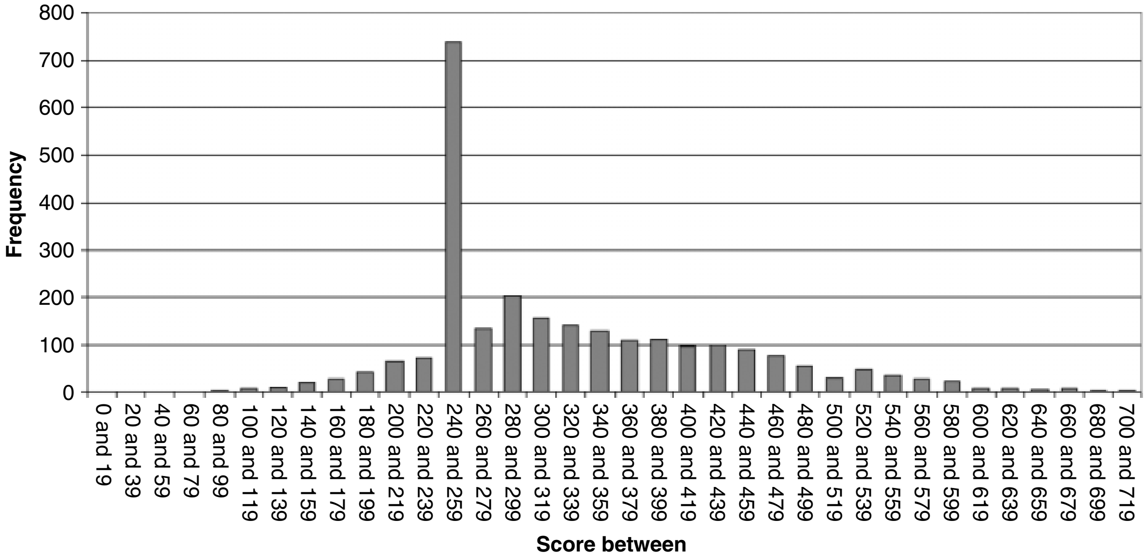

**Figure 2.** Frequency distribution of scores for potential targets in *Staphylococcus aureus*, based on the first scoring system used

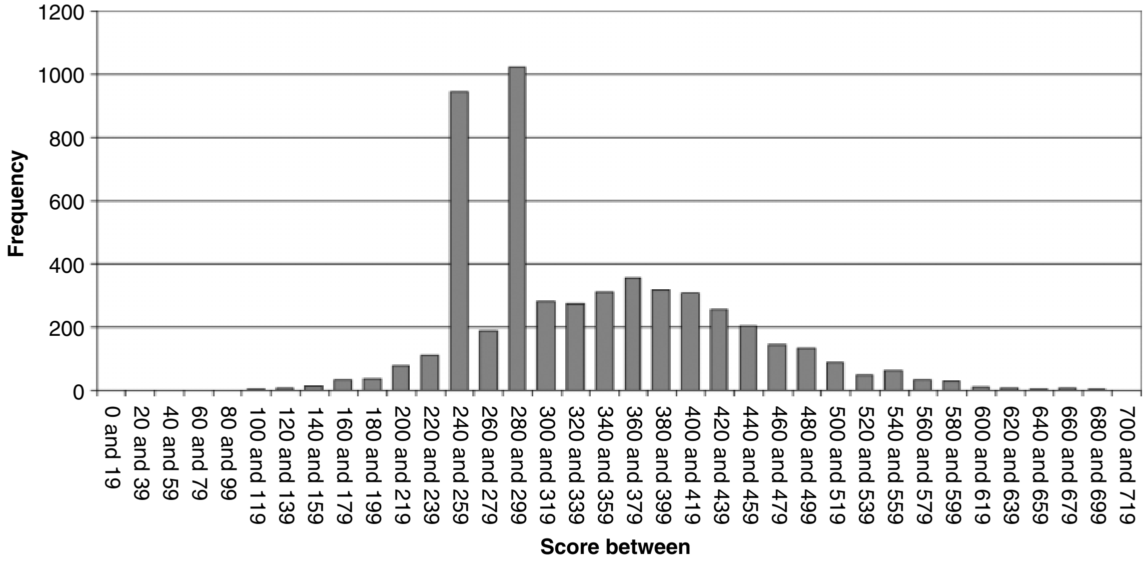

**Figure 3.** Frequency distribution of scores for potential targets in *Escherichia coli* O157:H7 EDL993, based on the first scoring system used

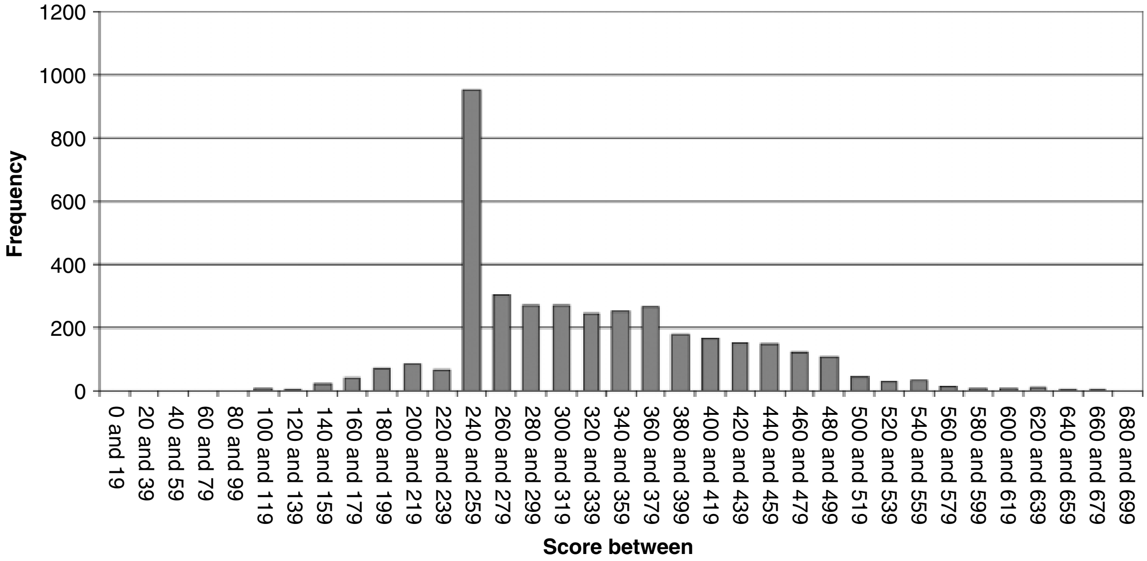

**Figure 4.** Frequency distribution of scores for potential targets in *Mycobacterium tuberculosis*, based on the first scoring system used the target to a human homologue which is important. Again, this is reflected in the scoring system, with the number of human homologues being less important than proximity. Of course the lack of any human homologues will bring other benefits, such as the reduced need for QSAR studies to find lead compounds that will selectively target only the bacterial version of a protein. In a similar way 'known function' and 'entry in PDB' are not crucial properties that a potential target must possess. They simply imply that something is already known about these targets which can be used as a jumping-off point for further investigation. 'Copy number' could be potentially important as, if a protein exists

Copyright © 2004 John Wiley & Sons, Ltd. 

in several copies and a drug targets only one copy, it is possible that a non-targeted copy could take over the function of the target, thus rendering the drug useless. However, it is likely that drugs which can disrupt the function of one copy will disrupt the function of both. Therefore, copy number is given a low/intermediate weighting in the scoring system. (Multiple paralogues also make the development of resistance much less likely.) The properties 'eukaryotic homologues' and 'mouse homologues' are not crucial, but again may make the process of drug development easier. A target without homologues in animal models will be more useful when it comes to carrying out animal trials. The lack of eukaryotic homologues may also allow the use of the target in developing drugs for livestock. The property 'homology to known target' is weighted fairly low in the first scoring system. Homology to a known target may imply something about the biochemical properties of a target which may be relevant to the drug design process. However, targets currently in use are not necessarily the best available, as problems with toxicity and resistance indicate. This study is also aimed at finding *novel* targets, which is why this property was not treated as being very important. The other scoring systems used are variants of this first scoring system, with the exception of the second, which simply treats all metrics as being equally important. The function of these scoring systems is to perturb the top-ranking targets, to weed out those targets which only perform well because of the vagaries of one scoring system.

We used sensitivity analyses on the top five ranking targets of *S. aureus* under the five different scoring systems (Figures 5–9) and also show the sensitivity analyses of lowest-ranking (Figure 10) and middle-ranking (Figure 11) targets from *S. aureus* under the first scoring system used. It can be seen that in most cases homology to virulence genes does not make much of a contribution to the total score of the top-ranking targets. This is generally because, unlike essential genes, virulence genes occur in only a limited spectrum of pathogens. As virulence genes tend to be more or less specific to one mode of pathogenicity, they do not occur in such a broad spectrum of organisms and consequently perform poorly in the metric 'distribution in bacteria' and 'distribution of homologues'. Similarly, when proteins *do* show homology to virulence genes in one pathogen, they often have no hits against virulence genes from other pathogens (e.g. see Figure 5).

The top-ranking targets in any scoring system also seem to have very similar sensitivity profiles, each generally being homologous to essential genes in all of the species used, having no homologues in humans and being well-conserved in a wide range of pathogens. Where target profiles differ is in the extent of the gene's distribution amongst pathogens, the extent of conservation (although not much), whether or not a

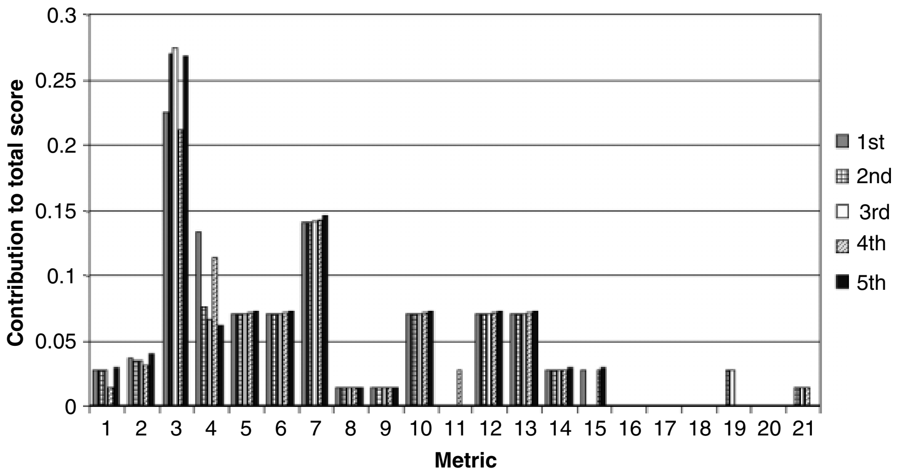

**Figure 5.** The contribution of each metric to the total score of the top five ranking targets in *Staphylococcus aureus*, based on the first scoring system used. Numbers refer to metrics described in Table 6. trmD is the top ranking (1st) target under this scoring system

Copyright © 2004 John Wiley & Sons, Ltd. 

structure is known, whether or not the gene is homologous to a known target, and whether or not the target is homologous to any virulence genes. From Figure 6 it seems that the in the profiles of the lowest-ranking targets the metric 'similarity to human homologue' becomes relatively more important. This is simply because these targets score so poorly on most of the other metrics. Many of these lower-ranking targets are hypothetical or unknown proteins which do not return many (if any) hits when submitted to BLAST, so are poorly characterized and score accordingly. Figure 7 shows that the profiles of middle-ranking targets vary. These targets score well on several criteria but poorly in others, such as the number of human homologues or homology to essential genes.

### High-ranking targets

A number of genes rank not only consistently highly under the five scoring systems used, but also appear in the top 20 targets in all three of the genomes used in this study (see Table 5). Of these,

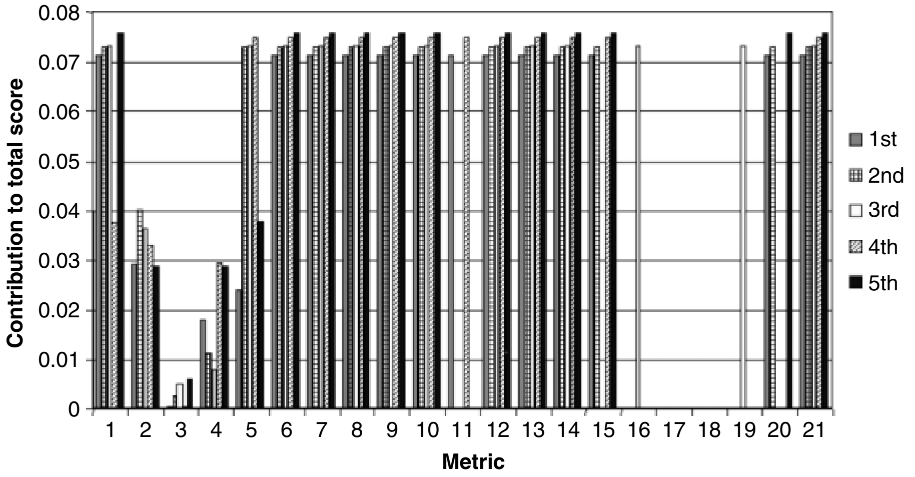

**Figure 6.** The contribution of each metric to the total score of the top five ranking targets in *Staphylococcus aureus*, based on the second scoring system used. Numbers refer to metrics described in Table 6

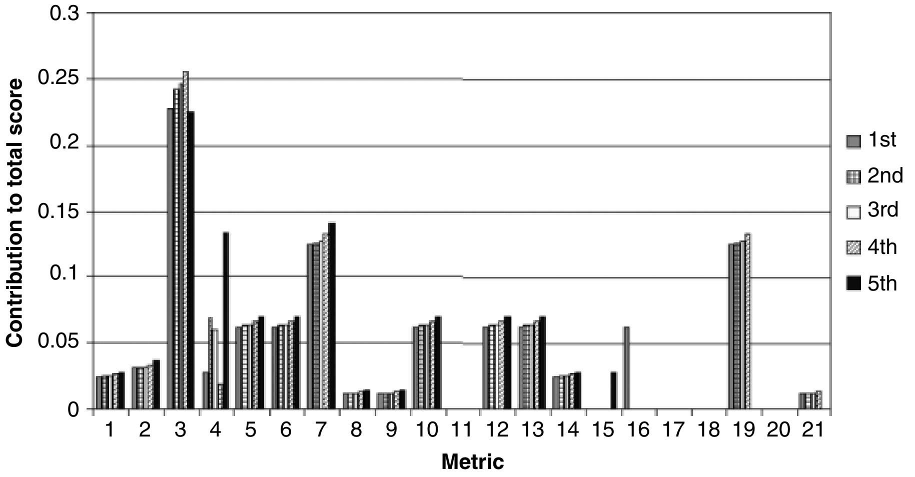

**Figure 7.** The contribution of each metric to the total score of the top five ranking targets in *Staphylococcus aureus*, based on the third scoring system used. Numbers refer to metrics described in Table 6. trmD ranks fifth from top under this scoring system

Copyright © 2004 John Wiley & Sons, Ltd. 

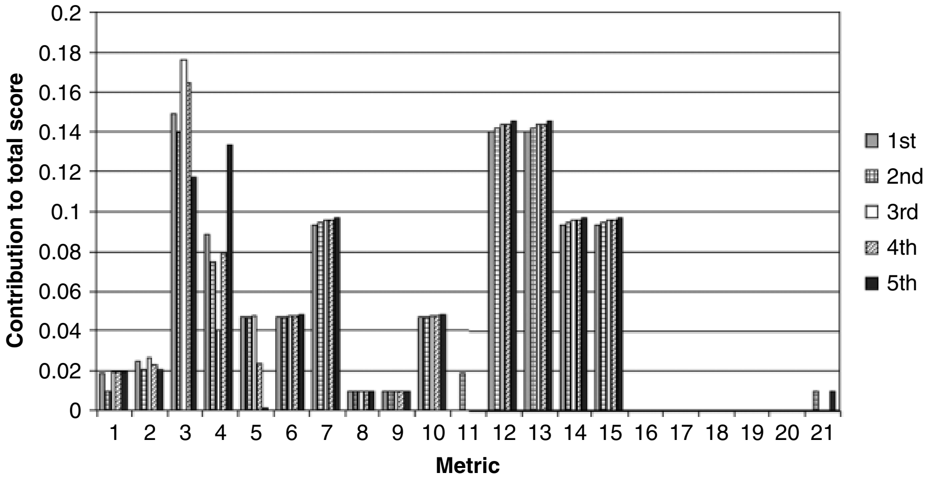

**Figure 8.** The contribution of each metric to the total score of the top five ranking targets in *Staphylococcus aureus*, based on the fourth scoring system used. Numbers refer to metrics described in Table 6. trmD is the top ranking target under this scoring system, while IF-1 (infA) ranks fifth

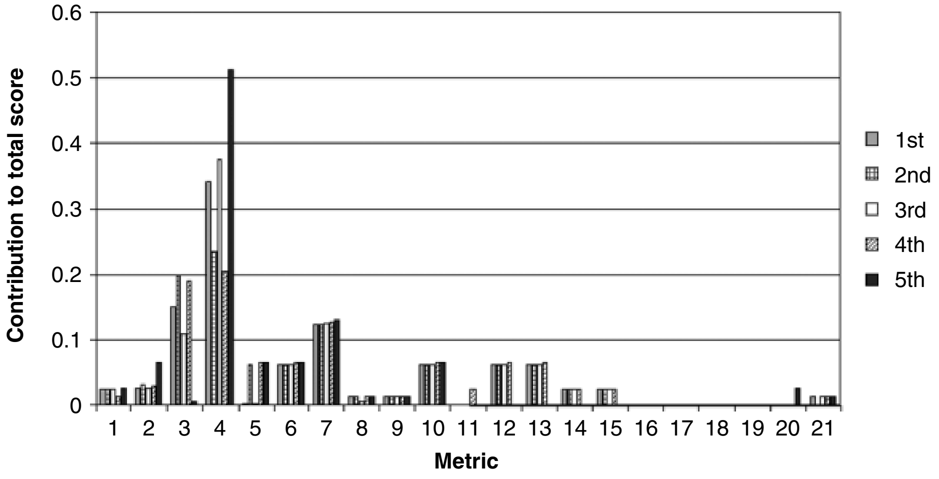

**Figure 9.** The contribution of each metric to the total score of the top five ranking targets in *Staphylococcus aureus*, based on the fifth scoring system used. Numbers refer to metrics described in Table 6. IF-1 is the top ranking target under this scoring system, while trmD ranks second

UDP-N-acetylglucosamine 1-carboxyvinyltransferase (murA) is a known target of fosfomycin, and the 30S ribosomal subunit protein S4 (rpsD) is a known target of tetracyclines. Of the others, dnaE, murC, murD and murE have been previously suggested as potential drug targets (Bouhss *et al.*, 1997; El Zoeiby *et al.*, 2003; Inoue *et al.*, 2001; Marmor *et al.*, 2001; Projan, 2002; Tanner *et al.*, 1996), and work carried out on murC, murD and murE has revealed effective inhibitors of these proteins (El Zoeiby *et al.*, 2003; Marmor *et al.*, 2001; Tanner *et al.*, 1996). The ribosome is of course currently heavily targeted by antibacterial drugs and suggestions for further work on ribosomal proteins have been made previously (Knowles and King, 1998). The existence of several known and previously suggested targets in the overall top 10 ranking in a sense validates this study, as it indicates that this method of target prioritization is indeed able to identify useful targets. There follows a brief discussion of the potential of some of the novel targets suggested.

Copyright © 2004 John Wiley & Sons, Ltd. 

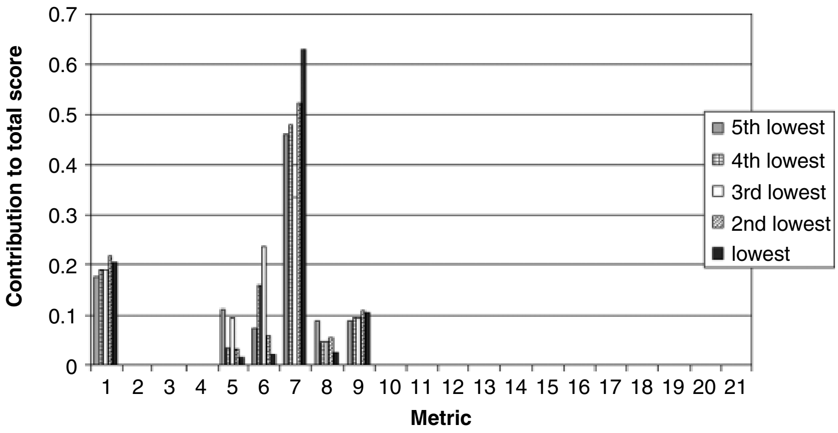

**Figure 10.** The contribution of each metric to the total score of the five lowest ranking targets in *Staphylococcus aureus* based on the first scoring system used. Numbers refer to metrics described in Table 6

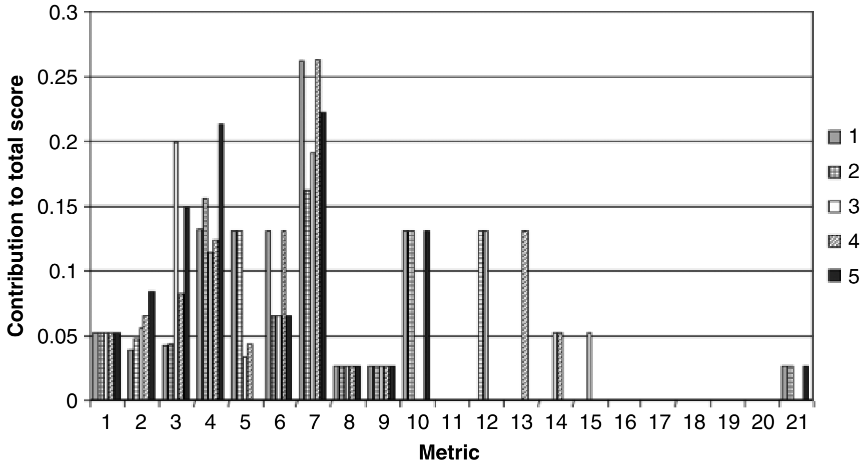

**Figure 11.** The contribution of each metric to the total score of five mid-ranking targets in *Staphylococcus aureus* based on the first scoring system used. Numbers refer to metrics described in Table 6

### tRNA methyltransferase (trmD)

tRNA methyltransferase (trmD) catalyses the transfer of a methyl group from S-adenosyl-L-methionine (AdoMet) to $G^{37}$ within a subset of bacterial tRNA species, which have a G residue at the 36th position (Ahn *et al.*, 2003). It is essential for the maintenance of the correct reading frame during translation. As an enzyme it is probably a better target than those requiring the inhibition of protein–protein interactions, although we note that progress in finding inhibitors of these is now being made (Oneyama *et al.*, 2002; Paulmurugan *et al.*, 2004).

The structure of the enzyme has been determined and is available from the Protein Data Bank (**http://www.rcsb.org/pdb/**) (Accession Nos 1UAJ, 1UAK, 1UAL and 1UAM) (Ahn *et al.*, 2003). The active site regions of the enzyme, which binds to AdoMet and tRNA, are known and are illustrated in Figure 12. It can be seen that these active site regions are highly conserved. This is encouraging from the point of view of designing a broad-spectrum drug to target this enzyme and also in terms of the reduced potential for resistant mutants emerging.

TrmD has, to our knowledge, never been recommended as an antibacterial drug target in the scientific literature, although it — along with

Copyright © 2004 John Wiley & Sons, Ltd. 

**Table 6.** Key to the metrics used

| Number | Description of metric |
|---|---|
| 1 | Copy number |
| 2 | Distribution of homologues (i.e. how many homologues in non-pathogens vs. pathogens?) |
| 3 | Distribution in pathogens (i.e. how many distinct pathogens is the gene present in, and in how many discrete non-pathogens?) |
| 4 | Conservation in pathogens |
| 5 | Number of homologues in eukaryotes |
| 6 | Number of human homologues |
| 7 | Similarity to human homologue |
| 8 | Number of mouse homologues |
| 9 | *Lactobacillus plantarum* homologues |
| 10 | Function known? |
| 11 | Homology to known target |
| 12 | Homology to essential gene in *Bacillus subtilis* |
| 13 | Homology to essential gene in *Escherichia coli* K12 |
| 14 | Homology to essential gene in *Mycobacterium tuberculosis* |
| 15 | Homology to essential gene in *Staphylococcus aureus* |
| 16 | Homology to virulence gene in *Bacillus anthracis* |
| 17 | Homology to virulence gene in *Escherichia coli* O157:H7 EDL993 |
| 18 | Homology to virulence gene in *Mycobacterium tuberculosis* |
| 19 | Homology to virulence gene in *Neisseria meningitidis* |
| 20 | Homology to virulence gene in *Staphylococcus aureus* |
| 21 | Homology to PDB entry |

a large batch of other bacterial proteins — has been patented on the basis of experiments in *S. aureus* in connection with its use as a drug target (United States Patent and Trademark Office **http://www.uspto.gov/**, Patent No. 6 187 541).

Figure 13 shows a neighbour-joining tree of trmD sequences in pathogenic and non-pathogenic bacteria. As can be seen, trmD is present in many pathogens, both Gram-positive and Gram-negative, indicating that it has the potential to be a very good broad-spectrum antibacterial drug target. The enzyme appears to function as a dimer (Ahn *et al.*, 2003), so one would probably seek to target the active site.

It is also important to know in which pathogens trmD is absent. Table 7 shows which of the pathogens (as defined by this study) are not known to possess trmD. It is important to note, however, that only a small number of these strains

**Table 7.** List of pathogenic bacteria (as defined by this study) which are not known to possess any homologues of the trmD gene under the expectation value used

| | |
|---|---|
| *Bacillus cereus* | *Neisseria gonorrhoeae* |
| *Bordetella pertussis* | *Neisseria meningitidis* serogroup C |
| *Brucella abortus* | *Propionibacterium acnes* |
| *Clostridium botulinum* | *Proteus mirabilis* |
| *Clostridium tetani** | *Providencia rettgeri* |
| *Corynebacterium diptheriae* | *Providencia stuartii* |
| *Enterococcus faecium* | *Salmonella cholerae-suis* |
| *Escherichia coli* 0111:H$^-$ | *Salmonella enteritidis* |
| *Escherichia coli* O127:H6 | *Shigella dysenteriae* |
| *Flavobacterium meningosepticum* | *Staphylococcus capitis* |
| *Francisella tularensis* | *Staphylococcus epidermidis** |
| *Haemophilus parainfluenzae* | *Staphylococcus saprophyticus* |
| *Klebsiella pneumoniae* | *Streptococcus agalactiae* |
| *Legionella pneumophila* | *Streptococcus agalactiae* serotype III* |
| *Moraxella catarrhalis* | *Streptococcus pyogenes* serotype M5* |
| *Moraxella lacunata* | *Vibrio parahaemolyticus** |
| *Mycoplasma fermentans* | *Wolinella recta* |
| *Mycoplasma hominis* | *Wolinella succinogenes* |
| | *Xanthomonas maltophila* |

* Species/strains whose genomes have been entirely sequenced.

**Figure 12.** (A) Alignment of trmD sequences found in pathogens. trmD sequences from *S. aureus*, *E. coli* O157:H7 EDL993 and *M. tuberculosis* were used as BLAST queries and the (non-redundant) hits from these searches were combined. BLAST was run with an expectation value of 0.01. A sequence from *Acinetobacter calcoaceticus* was removed, as it was considerably shorter than the others. Active site regions are highlighted by black boxes and labels show the function of the active site after Ahn *et al.* (2003). Sequences were aligned using ClustalX (Thompson *et al.*, 1997). Names are given as SwissProt style ID codes: TRMD_MYCPN is for trmD from *Mycoplasma pneumoniae*; MYCGE, *Mycoplasma genitalium*; UREUR, *Ureaplasma urealyticum*; BORBU, *Borrelia burgdorferi*; TREPA, *Treponema pallidum*; LISMO, *Listeria monocytogenes*; BACAN, *Bacillus anthracis*; ENTFA, *Enterococcus faecalis*; STRPY, *Streptococcus pyogenes*; STRPN, *Streptococcus pneumoniae*; STRA5, *Streptococcus agalactiae* serotype V; STRMU, *Streptococcus mutans*; STAAN, *Staphylococcus aureus* N315; CLOPE, *Clostridium perfringens*; MYCTU, *Mycobacterium tuberculosis*; MYCLE, *Mycobacterium leprae*; TROWH, *Tropheryma whipplei*; CHLTR, *Chlamydia trachomatis*; CHLMU, *Chlamydia muridarum*; CHLPN, *Chlamydia pneumoniae*; ECO57, *Escherichia coli* O157:H7 EDL993; SHIFL, *Shigella flexneri*; SALTY, *Salmonella typhimurium*; YERPE, *Yersinia pestis*; VIBVU, *Vibrio vulnificus*; VIBCH, *Vibrio cholerae*; PASMU, *Pasteurella multocida*; HAEIN, *Haemophilus influenzae*; HAEDU, *Haemophilus ducreyi*; PSAEA, *Pseudomonas aeruginosa*; NEIMA, *Neisseria meningitidis*; RICPR, for *Rickettsia prowazekii*; RICCN, *Rickettsia conorii*; BRUME, *Brucella melitensis*; CAMJE, *Campylobacter jejuni*; HELPY, *Helicobacter pylori*. (B) Alignment of selected trmD sequences from pathogenic bacteria and a human homologue (marked *). It can be seen that the human homologue only aligns with the bacterial proteins at the C-terminal end. See Figure 12 for further information

Copyright © 2004 John Wiley & Sons, Ltd. 

A

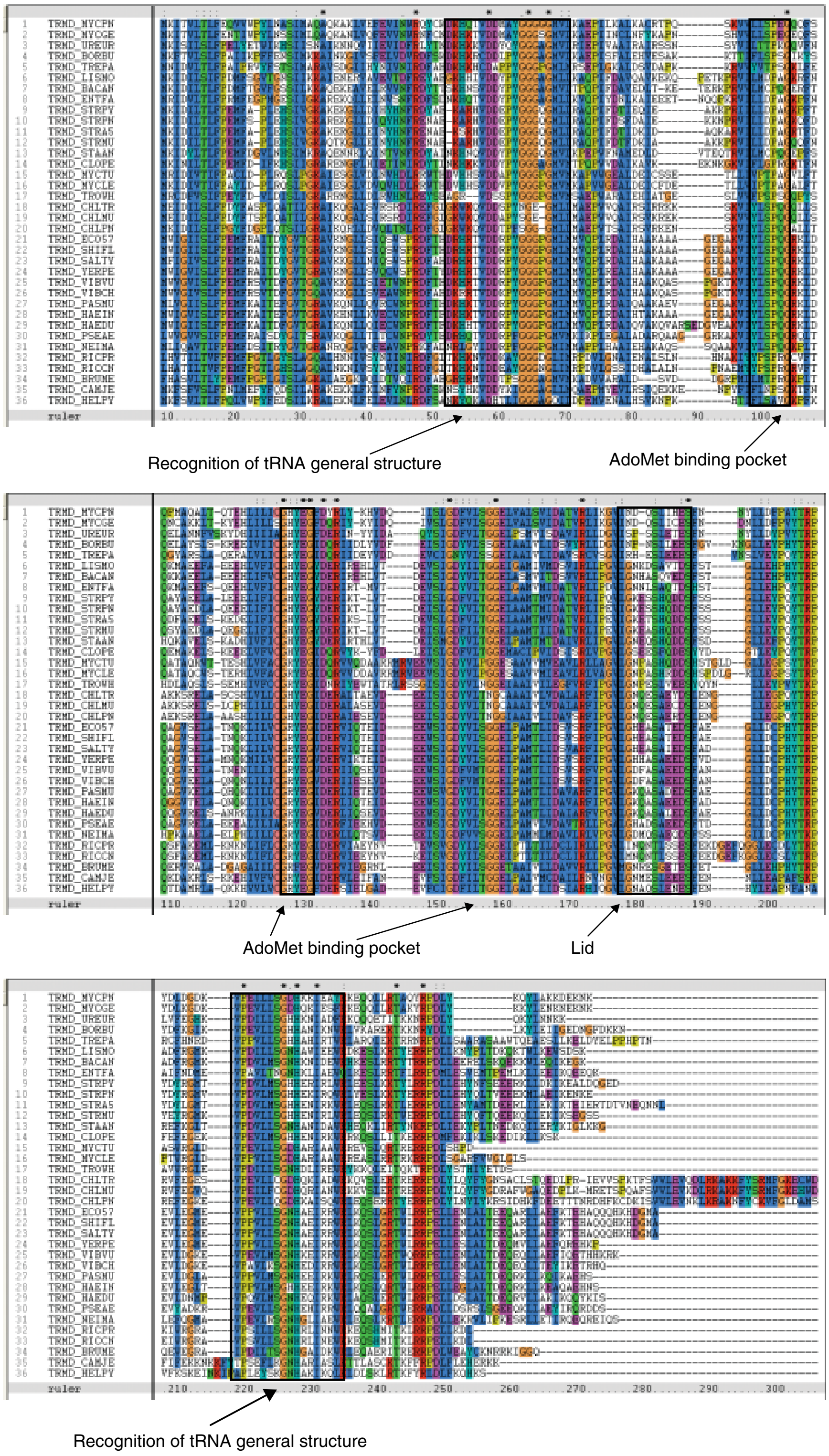

Copyright © 2004 John Wiley & Sons, Ltd. 

B

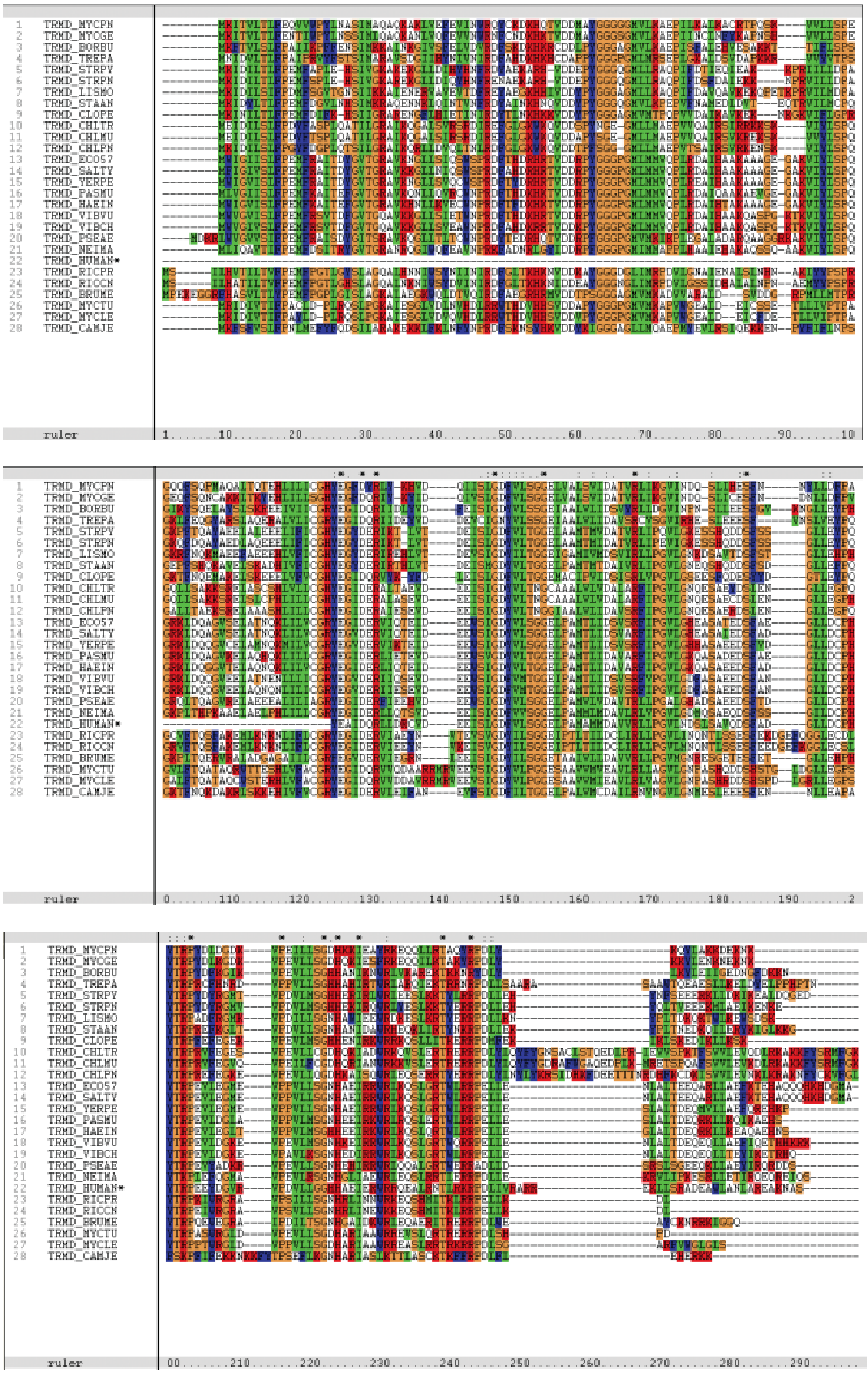

**Figure 12.** Continued

Copyright © 2004 John Wiley & Sons, Ltd. 

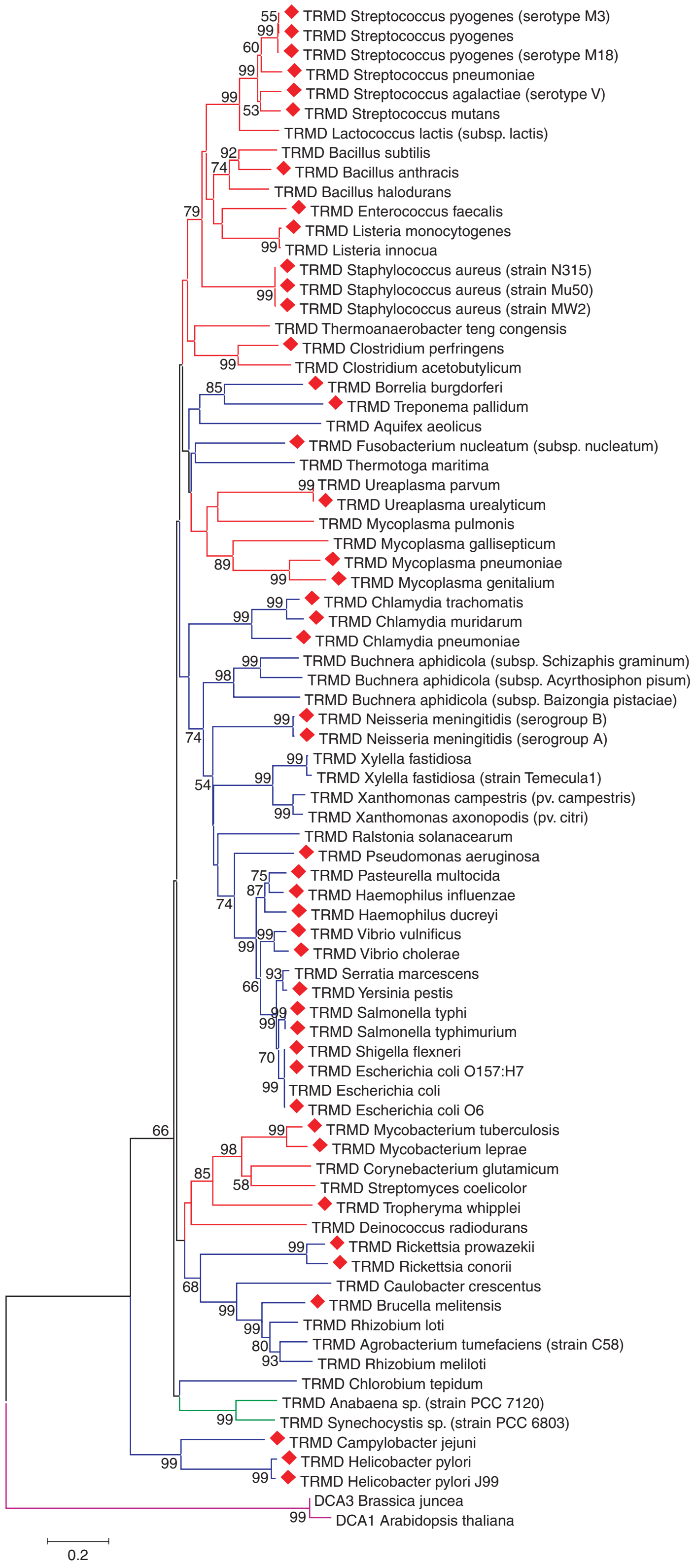

TRMD Streptococcus pyogenes (serotype M3)
TRMD Streptococcus pyogenes
TRMD Streptococcus pyogenes (serotype M18)
TRMD Streptococcus pneumoniae
TRMD Streptococcus agalactiae (serotype V)
TRMD Streptococcus mutans
TRMD Lactococcus lactis (subsp. lactis)
TRMD Bacillus subtilis
TRMD Bacillus anthracis
TRMD Bacillus halodurans
TRMD Enterococcus faecalis
TRMD Listeria monocytogenes
TRMD Listeria innocua
TRMD Staphylococcus aureus (strain N315)
TRMD Staphylococcus aureus (strain Mu50)
TRMD Staphylococcus aureus (strain MW2)
TRMD Thermoanaerobacter teng congensis
TRMD Clostridium perfringens
TRMD Clostridium acetobutylicum
TRMD Borrelia burgdorferi
TRMD Treponema pallidum
TRMD Aquifex aeolicus
TRMD Fusobacterium nucleatum (subsp. nucleatum)
TRMD Thermotoga maritima
TRMD Ureaplasma parvum
TRMD Ureaplasma urealyticum
TRMD Mycoplasma pulmonis
TRMD Mycoplasma gallisepticum
TRMD Mycoplasma pneumoniae
TRMD Mycoplasma genitalium
TRMD Chlamydia trachomatis
TRMD Chlamydia muridarum
TRMD Chlamydia pneumoniae
TRMD Buchnera aphidicola (subsp. Schizaphis graminum)
TRMD Buchnera aphidicola (subsp. Acyrthosiphon pisum)
TRMD Buchnera aphidicola (subsp. Baizongia pistaciae)
TRMD Neisseria meningitidis (serogroup B)
TRMD Neisseria meningitidis (serogroup A)
TRMD Xylella fastidiosa
TRMD Xylella fastidiosa (strain Temecula1)
TRMD Xanthomonas campestris (pv. campestris)
TRMD Xanthomonas axonopodis (pv. citri)
TRMD Ralstonia solanacearum
TRMD Pseudomonas aeruginosa
TRMD Pasteurella multocida
TRMD Haemophilus influenzae
TRMD Haemophilus ducreyi
TRMD Vibrio vulnificus
TRMD Vibrio cholerae
TRMD Serratia marcescens
TRMD Yersinia pestis
TRMD Salmonella typhi
TRMD Salmonella typhimurium
TRMD Shigella flexneri
TRMD Escherichia coli O157:H7
TRMD Escherichia coli
TRMD Escherichia coli O6
TRMD Mycobacterium tuberculosis
TRMD Mycobacterium leprae
TRMD Corynebacterium glutamicum
TRMD Streptomyces coelicolor
TRMD Tropheryma whipplei
TRMD Deinococcus radiodurans
TRMD Rickettsia prowazekii
TRMD Rickettsia conorii
TRMD Caulobacter crescentus
TRMD Brucella melitensis
TRMD Rhizobium loti
TRMD Agrobacterium tumefaciens (strain C58)
TRMD Rhizobium meliloti
TRMD Chlorobium tepidum
TRMD Anabaena sp. (strain PCC 7120)
TRMD Synechocystis sp. (strain PCC 6803)
TRMD Campylobacter jejuni
TRMD Helicobacter pylori
TRMD Helicobacter pylori J99
DCA3 Brassica juncea
DCA1 Arabidopsis thaliana
0.2

**Figure 13.** Neighbour-joining tree showing the distribution of trmD in pathogenic and non-pathogenic bacteria. Sequences were aligned in ClustalX (Thompson *et al*., 1997) (trmD from *Acinetobacter calcoaceticus* was removed, as it is considerably shorter than the other trmD sequences). The tree was created using Mega 2.1 using default parameters (Kumar *et al*., 2001). Branches leading to Gram-negative bacteria are coloured blue, those leading to Gram-positive bacteria in red, and those leading to Cyanobacteria in dark green. The tree is rooted using DCA3 from *Brassica juncea* and DCA1 from *Arabidopsis thaliana* as the outgroup (branches highlighted in purple). trmD sequences from bacteria treated as pathogenic by this study are marked with a red diamond. Numbers on branches show bootstrap support for groupings based on 100 replicates (values <50 are not shown). Scale bar shows number of substitutions per site have been extensively sequenced (those marked *). Thus, a copy of the gene may exist in these species/strains despite its absence from the databases. As can be seen, there are only a small number of species/strains which have been entirely sequenced and which do not possess a copy of trmD.

While there is a version of trmD in humans, the human version of the protein covers only covers the C-terminal end of the bacterial protein, as illustrated by Figure 12B. This may allow a selective drug to be developed which targets the bacterial but not the human version of the protein.

Another issue of importance in the development of a new drug is ease of assay development (Allsop, 1998). If a copy of a protein exists in yeast, then *in vitro* assay development is fairly straightforward via haploinsufficient phenotype (hp)-based strategies (Giaever *et al*., 1999). However, no trmD homologue exists in yeast, so an alternative strategy must be used here. Other assays will of course be necessary for cellular functional assays and analysis *in vivo*.

### Translation initiation factor IF-1 (infA)

Translation initiation factor IF-1 scores well on all criteria except for homology to virulence genes, homology to known targets and the number of homologues in eukaryotes. IF-1 is very well conserved in pathogens, and this contributes significantly to its high score.

The *precise* function of initiation factor IF-1 is unknown. However, it is known to be one of a number of factors essential for the establishment of the correct reading frame during translation (Dahlquist and Puglisi, 2000). It is therefore one determinant of translation accuracy. IF-1 is essential for cell viability and cells deficient in IF-1 exhibit few polysomes (Cummings and Hershey, 1994).

IF-1 is also well conserved across all the species/strains in which it is present, and thus a drug could be designed to attack this target in a broad spectrum of pathogens. A number of residues are identical in all sequences, perhaps indicating a high selection pressure against mutation at these positions. This is encouraging from the point of view of drug resistance. Any resistant strains arising through mutation of these residues could be severely attenuated compared to the wild-type form.

It has been observed that IF-1 contains a repeated sequence motif (S1-RM) which is also found in ribosomal protein S1 (whose function is to enhance translational initiation in Gram-negative bacteria) (Gribskov, 1992). Thus, a drug designed to target this motif could attack two different essential gene products at the same time, which would be highly advantageous from the point of view of drug resistance. This motif appears to be involved in RNA binding. However, this motif is also found in eukaryotic translation initiation factor $\alpha$-chains (**http://us.expasy.org/prosite/**) and several copies of this exist in humans. Figure 14 shows the sequences of IF-1 in pathogens aligned against the three eIF-1$\alpha$ sequences found in humans. A number of positions in the alignment are highly conserved in both humans and pathogens, so some skill may be required to develop a drug which targets pathogens but is not toxic to humans. This highlights the problem of using overall sequence similarity to determine the close functional and biochemical relatives of a gene product.

Nevertheless, there do exist cases of successful drugs which target proteins which are also present in humans, such as the antifungal strobilurins which target cytochrome $bc_1$ (Weber *et al*., 1990). It can also be seen from Figure 14 that there are a number of positions in the protein where all or most pathogenic sequences have one residue (or biochemically similar residues) but where human sequences possess a biochemically different residue. Therefore, there is still plenty of

Copyright © 2004 John Wiley & Sons, Ltd.

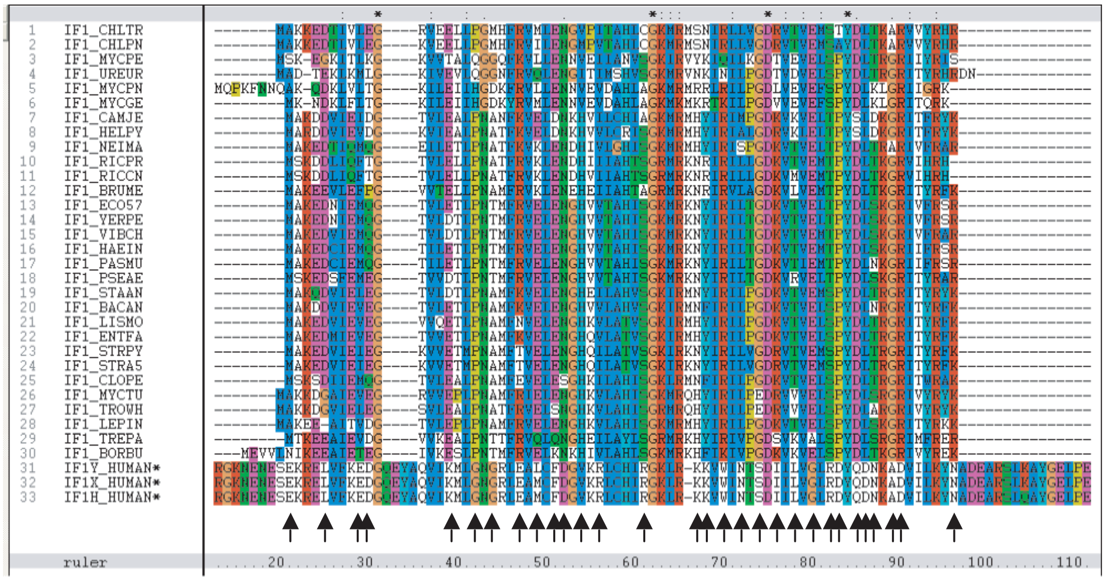

**Figure 14.** Alignment of IF1 proteins in pathogens and human eIF-1$\alpha$ sequences (bottom three sequences, marked *). Some identical sequences from close relatives of species/strains shown are omitted for ease of presentation. The alignment was created using ClustalX (Thompson *et al.*, 1997). As can be seen, a number of positions are highly conserved in both human and pathogen sequences. Black arrows mark positions where the *majority* of pathogens share the same or similar residues but where human sequences possess a different residue. IF1_CHLTR is for IF-1 from *Chlamydia trachomatis*; CHLPN, *Chlamydia pneumoniae*; MYCPE, *Mycoplasma penetrans*; UREUR, *Ureaplasma urealyticum*; MYCPN, *Mycoplasma pneumoniae*; MYCGE, *Mycoplasma genitalium*; CAMJE, *Campylobacter jejuni*; HELPY, *Helicobacter pylori*; NEIMA, *Neisseria meningitidis* serogroup A; RICPR, *Rickettsia prowazekii*; RICCN, *Rickettsia conorii*; BRUME, *Brucella melitensis*; ECO57 for *Escherichia coli* O157:H7 EDL993; YERPE, *Yersinia pestis*; VIBCH, *Vibrio cholerae*; HAEIN, *Haemophilus influenzae*; PASMU, *Pasteurella multocida*; PSEAE, *Pseudomonas aeruginosa*; STAAN, *Staphylococcus aureus* N315; BACAN, *Bacillus anthracis*; LISMO, *Listeria monocytogenes*; ENTFA, *Enterococcus faecalis*; STRPY, *Streptococcus pyogenes*; STRA5, *Streptococcus agalactiae* serotype V; CLOPE, *Clostridium perfringens*; MYCTU, *Mycobacterium tuberculosis*; TROWH, *Tropheryma whipplei*; LEPIN, *Leptospira interrogans*; TREPA, *Treponema pallidum*; BORBU, *Borrelia burgdorferi*; HUMAN, *Homo sapiens*

potential for the development of a drug which targets pathogens without interfering with the human form of the protein.

The structure of IF-1 has been determined and is available from the Protein Data Bank (**http://www.rcsb.org/pdb/**) (Accession Nos 1HRO and 1AH9). IF-1 is known to function as a monomer.

Figure 15 shows a phylogenetic tree of IF-1 and its homologues. It can be seen that the eukaryotic (chloroplast) sequences and those from bacteria can be split fairly well. Sequences from humans and pathogenic bacteria can be split easily. The sequences from pathogens and non-pathogens cannot be split, at least at the whole-sequence level, so it may prove impossible to develop a drug which targets IF-1 only in pathogens. This is not necessarily something to be overly concerned with, however, as curing the disease is more important than preserving the commensal flora.

There are a number of pathogens in which IF-1 has not been found (see Table 8). Again, this does not necessarily mean these pathogens do not possess a copy, as in many cases whole-genome sequencing has not been carried out. As can be seen, there are only a small number of species which have been entirely sequenced and in which IF-1 is absent.

### Conclusions

This study has used a simple but rational collection of criteria on which to rate bacterial gene products as potential broad-spectrum antibacterial drug targets. We assessed all the proteins from *S. aureus*, *E. coli* O157:H7 EDL993 and *M. tuberculosis* on criteria such as distribution, essentiality and involvement in virulence. All the proteins from each of these organisms were ranked in order of

Copyright © 2004 John Wiley & Sons, Ltd.

suitability as a potential drug target. It has been shown that although only a small proportion of gene products in any of the genomes would make useful drug targets, those which do rank highly do so fairly independently of the scoring system used. From these rankings it has been found that not only do a number of proteins rank highly under most or all of the different scoring systems used, but they also rank highly in all three genomes used. These targets have been described in some further detail and are left as suggestions for further in-depth analysis.

**Table 8.** List of pathogenic bacteria (as defined by this study) which do not possess any homologues of the infA gene under the expectation value used

| | |
|---|---|
| *Acinetobacter calcoaceticus* | *Neisseria meningitidis* serogroup B* |
| *Bacillus cereus* | *Neisseria meningitidis* serogroup C |
| *Bordetella pertussis* | *Propionibacterium acnes* |
| *Brucella abortus* | *Proteus mirabilis* |
| *Clostridium botulinum* | *Providencia rettgeri* |
| *Clostridium tetani** | *Providencia stuartii* |
| *Corynebacterium diptheriae* | *Salmonella cholerae-suis* |
| *Enterococcus faecium* | *Salmonella enteritidis* |
| *Escherichia coli* O111:H− | *Shigella dysenteriae* |
| *Escherichia coli* O127:H6 | *Staphylococcus capitis* |
| *Flavobacterium* | *Staphylococcus saprophyticus* |
| *meningosepticum* | *Streptococcus agalactiae* |
| *Francisella tularensis* | *Streptococcus agalactiae* serotype III* |
| *Haemophilus parainfluenzae* | *Streptococcus pyogenes* serotype M18* |
| *Klebsiella pneumoniae* | *Streptococcus pyogenes* serotype M5 |
| *Legionella pneumophila* | *Vibrio parahaemolyticus** |
| *Moraxella catarrhalis* | *Vibrio vulnificus** |
| *Moraxella lacunata* | *Wolinella recta* |
| *Mycoplasma fermentans* | *Wolinella succinogenes* |
| *Mycoplasma hominis* | *Xanthomonas maltophila* |
| *Neisseria gonorrhoeae* | |

* Species/strains whose genomes have been entirely sequenced.

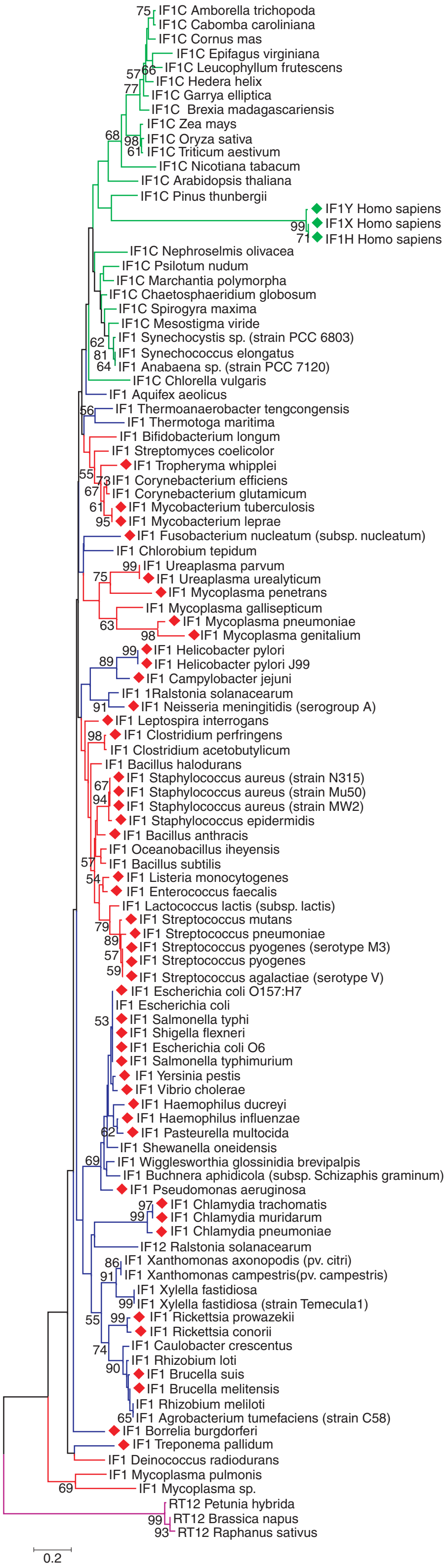

**Figure 15.** Neighbour-joining tree showing the distribution of infA in pathogenic and non-pathogenic bacteria and eukaryotes. Sequences were aligned in ClustalX (Thompson *et al.*, 1997). The tree was created using Mega 2.1 using default parameters (Kumar *et al.*, 2001). Branches leading to Gram-negative bacteria are coloured blue, those leading to Gram-positive bacteria in red, those leading to Cyanobacteria in dark green, and those leading to eukaryotes in light green. The tree is rooted using RT12 from *Petunia hybrida, Brassica napus* and *Raphanus sativus* as the outgroup (branches highlighted in purple). infA sequences from bacteria treated as pathogenic by this study are marked with a red diamond. Human sequences are marked with a green diamond. Numbers on branches show bootstrap support for groupings based on 100 replicates (values <50 are not shown). Scale bar shows number of substitutions per site

Copyright © 2004 John Wiley & Sons, Ltd. 

A number of known and previously suggested targets figure prominently in the overall top ten ranking. This shows that the methods used by this study can successfully identify targets which have the potential to be useful in the effective treatment of disease.

Through this study, a number of proteins have been suggested as entirely novel drug targets. However, the proof of these post-genomics methods will be in the successful development of a novel drug. Indeed, our scoring metrics made no attempt to consider a number of 'post-target-identification' criteria such as 'ease of assay'. Indeed, 'To date no antibacterial compounds identified by target-based screening have advanced into clinical testing, much less been used clinically to treat bacterial infections' (Projan, 2002). However, we argue that the two candidates identified (trmD and infA), as well as the methods exploited herein, provide promising strategies for the identification of novel antimicrobial targets, which can be analysed iteratively via hypothesis driven methods (Kell and Oliver, 2004). Whilst, of course, having a good target is hardly the same as having a good drug that inhibits it, developments of the sensitivity-based scoring approach could also be used in virtual screening, in the scoring of targets for 'druggability' (Hopkins and Groom, 2002; Zambrowicz and Sands, 2003) and of compounds for 'drug-like' qualities beyond the 'rule of 5' (Lipinski *et al.*, 2001). In this sense, multi-objective optimization methods (e.g. those of Coello Coello *et al.*, 2002; Dasgupta *et al.*, 1999; Deb, 2001; Knowles *et al.*, 2001; Zitzler, 1999) might also be used to advantage.

### Acknowledgements

DBK thanks the BBSRC, EPSRC, NERC and the RSC for financial support.

### References

Ahn HJ, Kim HW, Yoon HJ, *et al.* 2003. Crystal structure of tRNA(m1G37)methyltransferase: insights into tRNA recognition. *EMBO J* **22**: 2593–2603.

Alksne LE. 2002. Virulence as a target for antimicrobial chemotherapy. *Expert Opin Investig Drugs* **11**: 1149–1159.

Allsop A, Illingworth R. 2002. The impact of genomics and related technologies on the search for new antibiotics. *J Appl Microbiol* **92**: 7–12.

Allsop AE. 1998. New antibiotic discovery, novel screens, novel targets and impact of microbial genomics. *Curr Opin Microbiol* **1**: 530–534.

Altschul SF, Gish W, Miller W, Myers EW, Lipman DJ. 1990. Basic local alignment search tool. *J Mol Biol* **215**: 403–410.

Altschul SF, Madden TL, Schaffer AA. 1997. Gapped BLAST and PSI-BLAST: a new generation of protein database search programs. *Nucleic Acid Res* **25**: 3389–3402.

Bauer E, Kohavi R. 1999. An empirical comparison of voting classification algorithms: bagging, boosting and variants. *Machine Learning* **36**: 105–139.

Bouhss A, Mengin-Lecreulx D, Blanot D, van Heijenoort J, Parquet C. 1997. Invariant amino acids in the Mur peptide synthetases of bacterial peptidoglycan synthesis and their modification by site-directed mutagenesis in the UDP–MurNAc:L-alanine ligase from *Escherichia coli*. *Biochemistry* **36**: 11 556–11 563.

Brunder W, Khan AS, Hacker J, Karch H. 2001. Novel type of fimbriae encoded by the large plasmid of sorbitol-fermenting enterohemorrhagic *Escherichia coli* O157:H(–). *Infect Immun* **69**: 4447–4457.

Buysse JM. 2001. The role of genomics in antibacterial target discovery. *Curr Med Chem* **8**: 1713–1726.

Chittum HS, Champney WS. 1995. Erythromycin inhibits the assembly of the large ribosomal subunit in growing *Escherichia coli* cells. *Curr Microbiol* **30**: 273–279.

Chopra I, Hesse L, O'Neill AJ. 2002. Exploiting current understanding of antibiotic action for discovery of new drugs. *J Appl Microbiol* **92**(suppl): 4S–15S.

Coello Coello CA, van Veldhuizen DA, Lamont GB. 2002. *Evolutionary Algorithms for Solving Multi-objective Problems*. Kluwer Academic: Dordrecht.

Cummings HS, Hershey JW. 1994. Translation initiation factor IF1 is essential for cell viability in *Escherichia coli*. *J Bacteriol* **176**: 198–205.

Dahlquist KD, Puglisi JD. 2000. Interaction of translation initiation factor IF1 with the *E. coli* ribosomal A site. *J Mol Biol* **299**: 1–15.

Dasgupta P, Chakrabarti PP, DeSarkar SC. 1999. *Multiobjective Heuristic Search*. Vieweg: Braunschweig.

Davies J. 1994. Inactivation of antibiotics and the dissemination of resistance genes. *Science* **264**: 375–382.

Deb K. 2001. *Multi-objective Optimization Using Evolutionary Algorithms*. Wiley: Chichester.

Dietterich TG. 2000. An experimental comparison of three methods for constructing ensembles of decision trees: bagging, boosting, and randomization. *Machine Learning* **40**: 139–157.

Dougherty TJ, Barrett JF, Pucci MJ. 2002. Microbial genomics and novel antibiotic discovery: new technology to search for new drugs. *Curr Pharm Des* **8**: 1119–1135.

Dunman PM, Murphy E, Haney S, *et al.* 2001. Transcription profiling-based identification of *Staphylococcus aureus* genes regulated by the agr and/or sarA loci. *J Bacteriol* **183**: 7341–7353.

Egebjerg J, Douthwaite S, Garrett RA. 1989. Antibiotic interactions at the GTPase-associated centre within *Escherichia coli* 23S rRNA. *EMBO J* **8**: 607–611.

El Zoeiby A, Sanschagrin F, Levesque RC. 2003. Structure and function of the Mur enzymes: development of novel inhibitors. *Mol Microbiol* **47**: 1–12.

Fell DA. 1996. *Understanding the Control of Metabolism*. Portland Press: London.

Copyright © 2004 John Wiley & Sons, Ltd. 

Forsyth RA, Haselbeck RJ, Ohlsen KL, *et al.* 2002. A genome-wide strategy for the identification of essential genes in *Staphylococcus aureus*. *Mol Microbiol* **43**: 1387–1400.

Giaever G, Shoemaker DD, Jones TW, *et al.* 1999. Genomic profiling of drug sensitivities via induced haploinsufficiency. *Nature Genet* **21**: 278–283.

Gillies D. 1996. *Artificial Intelligence and Scientific Method.* Oxford University Press: Oxford.

Glass JI, Belanger AE, Robertson GT. 2002. *Streptococcus pneumoniae* as a genomics platform for broad-spectrum antibiotic discovery. *Curr Opin Microbiol* **5**: 338–342.

Gribskov M. 1992. Translational initiation factors IF-1 and eIF-2$\alpha$share an RNA-binding motif with prokaryotic ribosomal protein S1 and polynucleotide phosphorylase. *Gene* **119**: 107–111.

Hancock REW, Knowles D. 1998. Are we approaching the end of the antibiotic era? *Curr Opin Microbiol* **1**: 493–494.

Haney SA, Alksne LE, Dunman PM, Murphy E, Projan SJ. 2002. Genomics in anti-infective drug discovery — getting to endgame. *Curr Pharm Des* **8**: 1099–1118.

Hastie T, Tibshirani R, Friedman J. 2001. *The Elements of Statistical Learning: Data Mining, Inference and Prediction.* Springer-Verlag: Berlin.

Heinemann JA. 1999. How antibiotics cause antibiotic resistance. *Drug Discov Today* **4**: 72–79.

Heinrich R, Schuster S. 1996. *The Regulation of Cellular Systems.* Chapman & Hall: London.

Hoffmaster AR, Koehler TM. 1999. Control of virulence gene expression in *Bacillus anthracis*. *J Appl Microbiol* **87**: 279–281.

Hopkins AL, Groom CR. 2002. The druggable genome. *Nat Rev Drug Discov* **1**: 727–730.

Inoue R, Kaito C, Tanabe M, *et al.* 2001. Genetic identification of two distinct DNA polymerases, DnaE and PolC, that are essential for chromosomal DNA replication in *Staphylococcus aureus*. *Mol Genet Genom* **266**: 564–571.

Isaacson RE. 2002. Genomics and the prospects for the discovery of new targets for antibacterial and antifungal agents. *Curr Pharm Des* **8**: 1091–1098.

Ji Y. 2002. The role of genomics in the discovery of novel targets for antibiotic therapy. *Pharmacogenomics* **3**: 315–323.

Kell DB, Oliver SG. 2004. Here is the evidence, now what is the hypothesis? The complementary roles of inductive and hypothesis-driven science in the post-genomic era. *Bioessays* **26**: 99–105.

Kell DB, Westerhoff HV. 1986. Metabolic control theory: its role in microbiology and biotechnology. *FEMS Microbiol Rev* **39**: 305–320.

Knowles DJ, King F. 1998. The impact of bacterial genomics on antibacterial discovery. *Adv Exp Med Biol* **456**: 183–195.

Knowles JD, Watson RA, Corne DW. 2001. Reducing local optima in single-objective problems by multi-objectivization. In Proceedings of the 1st International Conference on Evolutionary Multi-criterion Optimization (EMO'01), E. Zitzler, *et al.* (eds). Springer: Berlin; 269–283.

Kobayashi K, Ehrlich SD, Albertini A, *et al.* 2003. Essential *Bacillus subtilis* genes. *Proc Natl Acad Sci USA* **100**: 4678–4683.

Koehler TM. 2002. *Bacillus anthracis* genetics and virulence gene regulation. *Curr Top Microbiol Immunol* **271**: 143–164.

Kornder JD. 2002. Streptomycin revisited: molecular action in the microbial cell. *Med Hypoth* **58**: 34–46.

Kumar S, Tamura K, Jakobsen IB, Nei M. 2001. MEGA2: molecular evolutionary genetics analysis software. *Bioinformatics* **17**: 1244–1245.

Lin AH, Murray RW, Vidmar TJ, Marotti KR. 1997. The oxazolidinone eperezolid binds to the 50S ribosomal subunit and competes with binding of chloramphenicol and lincomycin. *Antimicrob Agents Chemother* **41**: 2127–2131.

Lipinski CA, Lombardo F, Dominy BW, Feeney PJ. 2001. Experimental and computational approaches to estimate solubility and permeability in drug discovery and development settings. *Adv Drug Deliv Rev* **46**: 3–26.

Marmor S, Petersen CP, Reck F, *et al.* 2001. Biochemical characterization of a phosphinate inhibitor of *Escherichia coli* MurC. *Biochemistry* **40**: 12 207–12 214.

McDevitt D, Rosenberg M. 2001. Exploiting genomics to discover new antibiotics. *Trends Microbiol* **9**: 611–617.

Mitchell TM. 1997. *Machine Learning*. McGraw Hill: London.

Mjolsness E, DeCoste D. 2001. Machine learning for science: state of the art and future prospects. *Science* **293**: 2051–2055.

Neu HC, Gootz TD. 1996. Antimicrobial chemotherapy. In *Medical Microbiology*, Baron S (ed.). University of Texas Medical Branch.

Oneyama C, Nakano H, Sharma SV. 2002. UCS15A, a novel small molecule, SH3 domain-mediated protein–protein interaction blocking drug. *Oncogene* **21**: 2037–2050.

Paulmurugan R, Massoud TF, Huang J, Gambhir SS. 2004. Molecular imaging of drug-modulated protein–protein interactions in living subjects. *Cancer Res* **64**: 2113–2119.

Payne DJ, Holmes DJ, Rosenberg M. 2001a. Delivering novel targets and antibiotics from genomics. *Curr Opin Investig Drugs* **2**: 1028–1034.

Payne DJ, Warren PV, Holmes DJ, Ji Y, Lonsdale JT. 2001b. Bacterial fatty-acid biosynthesis: a genomics-driven target for antibacterial drug discovery. *Drug Discov Today* **6**: 537–544.

Projan SJ. 2002. New (and not so new) antibacterial targets — from where and when will the novel drugs come? *Curr Opin Pharmacol* **2**: 513–522.

Sassetti CM, Boyd DH, Rubin EJ. 2003. Genes required for mycobacterial growth defined by high density mutagenesis. *Mol Microbiol* **48**: 77–84.

Schmid MB. 1998. Novel approaches to the discovery of antimicrobial agents. *Curr Opin Chem Biol* **2**: 529–534.

Schnappinger D, Hillen W. 1996. Tetracyclines: antibiotic action, uptake, and resistance mechanisms. *Arch Microbiol* **165**: 359–369.

Sharma VK, Dean-Nystrom EA. 2003. Detection of enterohemorrhagic *Escherichia coli* O157:H7 by using a multiplex real-time PCR assay for genes encoding intimin and Shiga toxins. *Vet Microbiol* **93**: 247–260.

Spaltmann F, Blunck M, Ziegelbauer K. 1999. Computer-aided target selection-prioritizing targets for antifungal drug discovery. *Drug Discov Today* **4**: 17–26.

Stephenson K, Hoch JA. 2002. Two-component and phosphorelay signal-transduction systems as therapeutic targets. *Curr Opin Pharmacol* **2**: 507–512.

Stephenson K, Hoch JA. 2004. Developing inhibitors to selectively target two-component and phosphorelay signal transduction systems of pathogenic microorganisms. *Curr Med Chem* **11**: 765–773.

Copyright © 2004 John Wiley & Sons, Ltd. 

Struelens MJ. 1998. The epidemiology of antimicrobial resistance in hospital acquired infections: problems and possible solutions. *Br Med J* **317**: 652–654.

Stuber K, Frey J, Burnens AP, Kuhnert P. 2003. Detection of type III secretion genes as a general indicator of bacterial virulence. *Mol Cell Probes* **17**: 25–32.

Sun YH, Bakshi S, Chalmers R, Tang CM. 2000. Functional genomics of *Neisseria meningitidis* pathogenesis. *Nature Med* **6**: 1269–1273.

Tanner ME, Vaganay S, van Heijenoort J, Blanot D. 1996. Phosphinate inhibitors of the D-glutamic acid-adding enzyme of peptidoglycan biosynthesis. *J Org Chem* **61**: 1756–1760.

Terstappen GC, Reggiani A. 2001. *In silico* research in drug discovery. *Trends Pharmacol Sci* **22**: 23–26.

Thompson JD, Gibson TJ, Plewniak F, Jeanmougin F, Higgins DG. 1997. The CLUSTAL_X windows interface: flexible strategies for multiple sequence alignment aided by quality analysis tools. *Nucleic Acids Res* **25**: 4876–4882.

Triccas JA, Gicquel B. 2000. Life on the inside: probing *Mycobacterium tuberculosis* gene expression during infection. *Immunol Cell Biol* **78**: 311–317.

Wang G, Clark CG, Rodgers FG. 2002. Detection in *Escherichia coli* of the genes encoding the major virulence factors, the genes defining the O157:H7 serotype, and components of the type 2 Shiga toxin family by multiplex PCR. *J Clin Microbiol* **40**: 3613–3619.

Weber W, Anke T, Bross M, Steglich W. 1990. Strobilurin D and strobilurin F: two new cytostatic and antifungal (E)-$\beta$-methoxyacrylate antibiotics from *Cyphellopsis anomala* (1). *Planta Med* **56**: 446–450.

Willins DA, Kessler M, Walker SS, Reyes GR, Cottarel G. 2002. Genomics strategies for antifungal drug discovery — from gene discovery to compound screening. *Curr Pharm Des* **8**: 1137–1154.

Zambrowicz BP, Sands AT. 2003. Knockouts model the 100 best-selling drugs — will they model the next 100? *Nature Rev Drug Discov* **2**: 38–51.

Zitzler E. 1999. *Evolutionary Algorithms for Multiobjective Optimization: Methods and Applications*. Shaker Verlag: Aachen.

Copyright © 2004 John Wiley & Sons, Ltd. 
